# The RNA-binding protein CELF1 targets ATG5 to regulate autophagy and promote drug resistance in acute myeloid leukemia

**DOI:** 10.1038/s41419-025-07926-0

**Published:** 2025-08-08

**Authors:** Xiaoyan Li, Qiyi Qian, Juejiashan Li, Lu Zhang, Lifang Wang, Dongsheng Huang, Qiuran Xu, Wenhu Chen

**Affiliations:** 1 School of Basic Medical Sciences & Forensic Medicine, Hangzhou Medical College, Hangzhou, China; 2 School of Life Sciences, Zhejiang Chinese Medical University, Hangzhou, China; 3 Zhejiang Key Laboratory of Tumor Molecular Diagnosis and Individualized Medicine, Zhejiang Provincial People’s Hospital, Affiliated People’s Hospital, Hangzhou Medical College, Hangzhou, Zhejiang China

**Keywords:** Cancer, Prognostic markers

## Abstract

Acute myeloid leukemia (AML) is a blood cancer characterized by uncontrolled growth of myeloid cells. Overcoming AML treatment resistance, particularly to anthracycline-based drugs like doxorubicin (ADR), poses a challenge. This study investigated the role of CELF1, an RNA-binding protein, in ADR resistance and autophagy regulation in AML. CELF1 expression was elevated in multiple tumor types, including AML. AML cell lines exhibit varying levels of CELF1 expression, with drug-resistant cell lines showing higher CELF1 expression compared to parental cells. CELF1 knockdown reduced drug resistance, promoted cell death, and inhibited autophagy. Mechanistic analysis identified ATG5 as a potential CELF1-regulated target gene, with CELF1 knockdown reducing ATG5 expression and mRNA decay. These findings indicate that targeting CELF1 could overcome ADR resistance in AML by modulating autophagy through ATG5 regulation, highlighting its clinical significance as a therapeutic target for enhancing ADR response in AML.

## Introduction

Acute Myeloid Leukemia (AML) is a type of blood cancer characterized by abnormal proliferation and differentiation of myeloid cells, with high mortality rates primarily affecting older adults [1]. The overall 5-year survival rate is less than 40% [2]. The hallmark features of AML include immature myeloid cell heterogeneity and rapid growth rate, leading to disrupted normal hematopoiesis. Despite the development of novel agents and improved treatment strategies, relapse and drug resistance remain major hurdles towards achieving optimal cure rates for AML patients [3]. Abnormalities in key genes during the progression of the disease may be crucial drivers of AML development and drug resistance [4]. Therefore, exploring novel biomarkers and gaining a deeper understanding of their impact on the biological characteristics of AML and the molecular mechanisms of clinical resistance could potentially enhance treatment outcomes and survival rates for AML patients.

The occurrence and development of AML are largely influenced by the regulation of RNA-binding proteins (RBP). Research has shown that aberrant expression of RBPs is often associated with enhanced transcriptional and post-transcriptional mechanisms promoting cancer progression [5, 6]. CELF1, a member of the CUGBP Elav-like family (CELF) of RNA-binding proteins, was initially identified as an RBP binding to the CUG triplet repeat sequence within muscle atrophy kinase mRNA. It plays a crucial role in the pathogenesis of Myotonic dystrophy (dystrophia myotonica, DM) [7, 8]. Further studies have revealed that CELF1 binds specifically to certain RNA elements and regulates numerous post-transcriptional processes such as alternative splicing, translational control, and mRNA decay regulation, thereby contributing to the progression of various human diseases, including cancer [9–11]. However, research on CELF1 in AML has not been reported. Our study found that CELF1 is abnormally highly expressed in AML cells, with a more pronounced expression observed in Adriamycin-resistant (ADR) cells. Further exploration revealed that CELF1 may be involved in the regulation of autophagy in ADR resistance, but the key molecular mechanisms remain to be elucidated.

The development of chemotherapy resistance in hematological malignancies poses a significant challenge to clinical treatment, representing one of the major obstacles in tumor therapy [12, 13]. Research has demonstrated that autophagy plays a crucial role in tumorigenesis and progression [14]. Specifically, autophagy can eliminate mutated cells, damaged cellular organelles, and genomic instability during early stages of tumor formation, thereby inhibiting tumor initiation [15]. In established tumors, autophagy is enhanced as a response to nutrient deprivation, energy insufficiency, hypoxic stress, and chemotherapy-induced damage, allowing cancer cells to continue growth and dissemination without destruction, ultimately leading to the induction of acquired resistance [16–18]. In certain hematological malignancies, autophagy has been found to be a key mechanism that enables tumor cells to evade immune surveillance and killing [19–21]. Moreover, after exposure to chemotherapeutic agents, autophagy can be activated in these cancer cells, resulting in the development of drug resistance [22–24].

This study discovered that CELF1 specifically binds to the mRNA of ATG5, a critical gene involved in autophagy regulation, and promotes its expression in ADR-resistant acute myeloid leukemia (AML) cells. This activation of protective autophagy enhances the AML cells’ resistance to ADR treatment. Furthermore, we established an in vivo model system to validate CELF1’s regulatory role on ATG5-mediated autophagy as a tumor-suppressive mechanism. Therefore, targeting CELF1 specifically or inhibiting ATG5-mediated autophagy has significant implications for further translational research and clinical application in AML treatment.

## Results

### CELF1 is a potential target for ADR resistance in AML cells

**Fig. 1:**
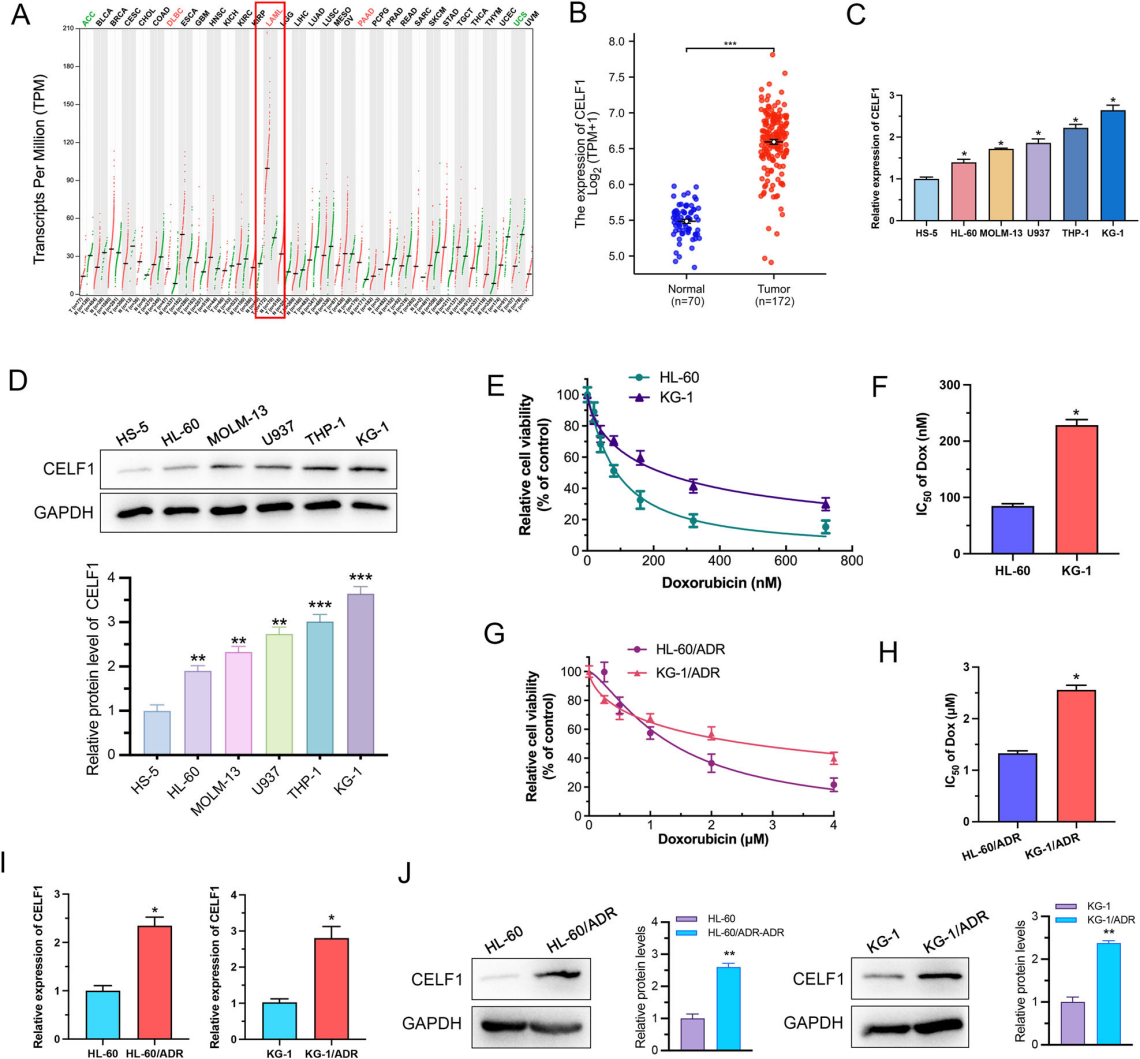
CELF1 is overexpressed in in AML drug-resistant cell lines. **A**, **B** Pan-cancer expression of CELF1 between tumor tissues and normal tissues from TCGA. **C**, **D** RT-qPCR and Western Blot evaluate CELF1 expression across diverse AML cell lines. **E**, **F** The IC_50_ values and inhibitory curve in KG-1 and HL-60 cells with ADR tested. **G**, **H** The IC_50_ values and inhibitory curve in KG-1/ADR and HL-60/ADR cells with ADR tested. **I**, **J** RT-qPCR and Western Blot analysis of CELF1 expression in AML drug-resistant cells compared to parental cells.

Firstly, we conducted a pan-cancer analysis of CELF1 expression using TCGA data, revealing significantly elevated levels of CELF1 across various tumors, with the most notable difference observed in AML (Fig. 1A, B). Clinical baseline data indicates a significant correlation between high CELF1 expression and lower classifications within the FAB system (M0&M1&M2), while no significant correlations were found with Bone Marrow Blasts proportion, Age, Cytogenetic risk, and FLT3 mutation (Table 1). Then, We selected five commonly used AML cell lines (HL-60, MOLM-13, U937, THP-1, and KG-1) to investigate CELF1 expression levels using RT-qPCR and Western Blot. The results indicate that the levels of CELF1 mRNA and protein expression in AML cell lines are significantly higher than those in HS-5. Furthermore, there are variations in CELF1 expression levels among the different cell lines, with KG-1 exhibiting the highest expression and HL-60 the lowest (Fig. 1C, D). To validate the relationship between CELF1 expression and ADR resistance, we conducted CCK-8 assays on KG-1 and HL-60 cells. Compared to HL-60 cells, KG-1 cells demonstrated significant resistance to ADR treatment (Fig. 1E). Notably, the IC_50_ values of ADR in KG-1 cells were substantially higher than those observed in HL-60 cells (228.6 ± 8.3 nM vs. 84.21 ± 3.03 nM) (Fig. 1F). We established two ADR-resistant cell lines, KG-1/ADR (2558 ± 86 nM) and HL-60/ADR (1328 ± 41 nM), which exhibited increased IC_50_ values compared to their parental cells. Specifically, the IC_50_ values of KG-1/ADR and HL-60/ADR cells increased by ~10-fold (Fig. 1G, H). RT-qPCR and Western Blot analysis revealed that CELF1 expression was significantly upregulated in both resistant cell lines (Fig. 1I, J). This finding suggests a potential clinical relevance for targeting CELF1 to overcome ADR resistance in AML.

**Table 1:** Correlations between CELF1 expression and clinicopathologic features of AML patients.

Characteristics	Low expression of CELF1	High expression of CELF1	*P* value
*n*	75	75	
Gender, *n* (%)			0.412
Female	36 (24%)	31 (20.7%)	
Male	39 (26%)	44 (29.3%)	
Race, *n* (%)			0.979
Asian&Black or African American	7 (4.7%)	7 (4.7%)	
White	68 (45.6%)	67 (45%)	
FAB classifications, *n* (%)			0.022*
M0&M1&M2	36 (24.2%)	52 (34.9%)	
M3	9 (6%)	5 (3.4%)	
M4&M5&M6&M7	30 (20.1%)	17 (11.4%)	
BM blasts(%), *n* (%)			0.867
≤20	29 (19.3%)	30 (20%)	
>20	46 (30.7%)	45 (30%)	
Age, *n* (%)			0.408
≤60	46 (30.7%)	41 (27.3%)	
>60	29 (19.3%)	34 (22.7%)	
WBC count(×10^9/L), *n* (%)			0.933
≤20	38 (25.5%)	38 (25.5%)	
> 20	36 (24.2%)	37 (24.8%)	
Cytogenetic risk, *n* (%)			0.487
Favorable	15 (10.1%)	15 (10.1%)	
Intermediate/normal	44 (29.7%)	38 (25.7%)	
Poor	15 (10.1%)	21 (14.2%)	
FLT3 mutation, *n* (%)			0.432
Negative	52 (35.6%)	49 (33.6%)	
Positive	20 (13.7%)	25 (17.1%)	

Clinical data were derived from The Cancer Genome Atlas (TCGA) AML cohort.

*AML* acute myelogenous leukemia, *FAB* French-American-British classification, *WBC* White Blood Cell.

### CELF1 knockdown promotes drug sensitivity and reduces autophagy in AML cells

In the treatment of acute myeloid leukemia (AML), the development of drug resistance remains a major cause of chemotherapy failure and disease relapse. Recent studies suggest that autophagy, a critical regulatory mechanism of cellular metabolic homeostasis, plays a pivotal role in AML drug resistance. Therefore, We performed Western blot to assess the expression of three key autophagy markers (p62, Beclin-1, LC3-II/LC3-I) to evaluate changes in autophagy levels in drug-resistant cells and their parental counterparts. The results revealed increased LC3-II/LC3-I ratios, decreased p62 protein levels, and elevated Beclin1 protein levels in HL-60/ADR and KG-1/ADR cells compared to HL-60 and KG-1 cells, indicating significantly enhanced autophagic activity in the resistant cells (Supplementary Fig. S1A). Treatment with the autophagy inhibitor Bafilomycin A1 (BafA1) markedly suppressed autophagic activation in HL-60/ADR and KG-1/ADR cells (Supplementary Fig. S1B). Tandem GFP-mRFP-LC3 fluorescence analysis showed that BafA1 treatment decreased the number of yellow and red puncta in HL-60/ADR and KG-1/ADR cells, confirming its role in reversing autophagy activation in drug-resistant cells (Supplementary Fig. S1C). Furthermore, CCK-8 assays demonstrated that BafA1 reduced the IC_50_ values of ADR in both resistant cell lines (Supplementary Fig. S1D).

**Fig. 2:**
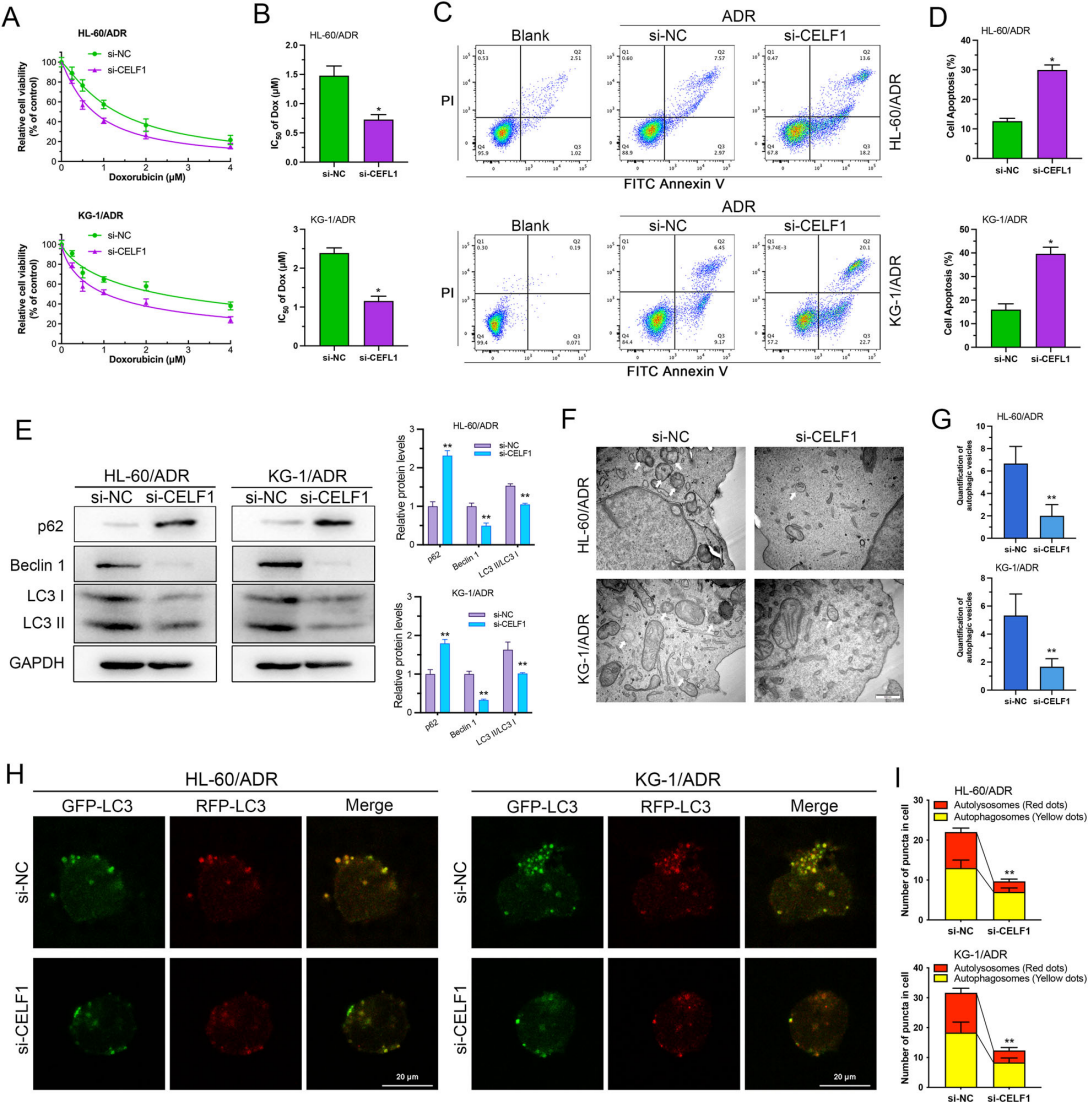
CELF1 knockdown promotes cell apoptosis and inhibits autophagy. **A**, **B** CCK8 assess IC_50_ values and inhibition curves after CELF1 knockdown in KG-1/ADR and HL-60/ADR cells. **C**, **D** Flow cytometry analysis of apoptosis in cells following CELF1 knockdown. **E** Western blot analysis of autophagy markers in cells after CELF1 knockdown. **F**, **G** Autophagic organelles visualized by transmission electron microscopy in cells after CELF1 knockdown. **H**, **I** Tandem mRFP-GFP-LC3 fluorescence assay performed in CELF1 knockdown cells to monitor autophagic flux.

To validate the role of CELF1 in AML drug resistance, we used siRNA to knockdown CELF1 expression in KG-1/ADR and HL-60/ADR cells, with the silencing efficiency demonstrated in Supplementary Fig. S2A, B. CCK-8 assay results indicated that downregulation of CELF1 significantly diminished the drug-resistant phenotype (Fig. 2A, B). Furthermore, flow cytometry results showed that CELF1 knockdown significantly increased ADR-induced cell apoptosis (Fig. 2C, D). Results of Western blot revealed a significant upregulation of p62 expression and a marked decrease in the ratio of LC3-II/LC3-I and Beclin-1 expression after CELF1 knockdown (Fig. 2E), indicating that CELF1 knockdown can inhibit autophagy levels. Transmission electron microscopy images confirmed these findings, showing a significant reduction in the number of autophagosomes in CELF1 knockdown KG-1/ADR and HL-60/ADR cells compared to the control group (Fig. 2F, G). Additionally, we conducted tandem mRFP-GFP-LC3 fluorescence analysis. The results indicated that knockdown of CELF1 resulted in a reduction in the number of yellow and red puncta in HL-60/ADR and KG-1/ADR cells, effectively block autophagic flux (Fig. 2H, I). The above results indicate that CELF1 knockdown promotes AML cell apoptosis and inhibits autophagy.

### CELF1 enhances AML cell sensitivity to ADR through inhibition of autophagy

**Fig. 3:**
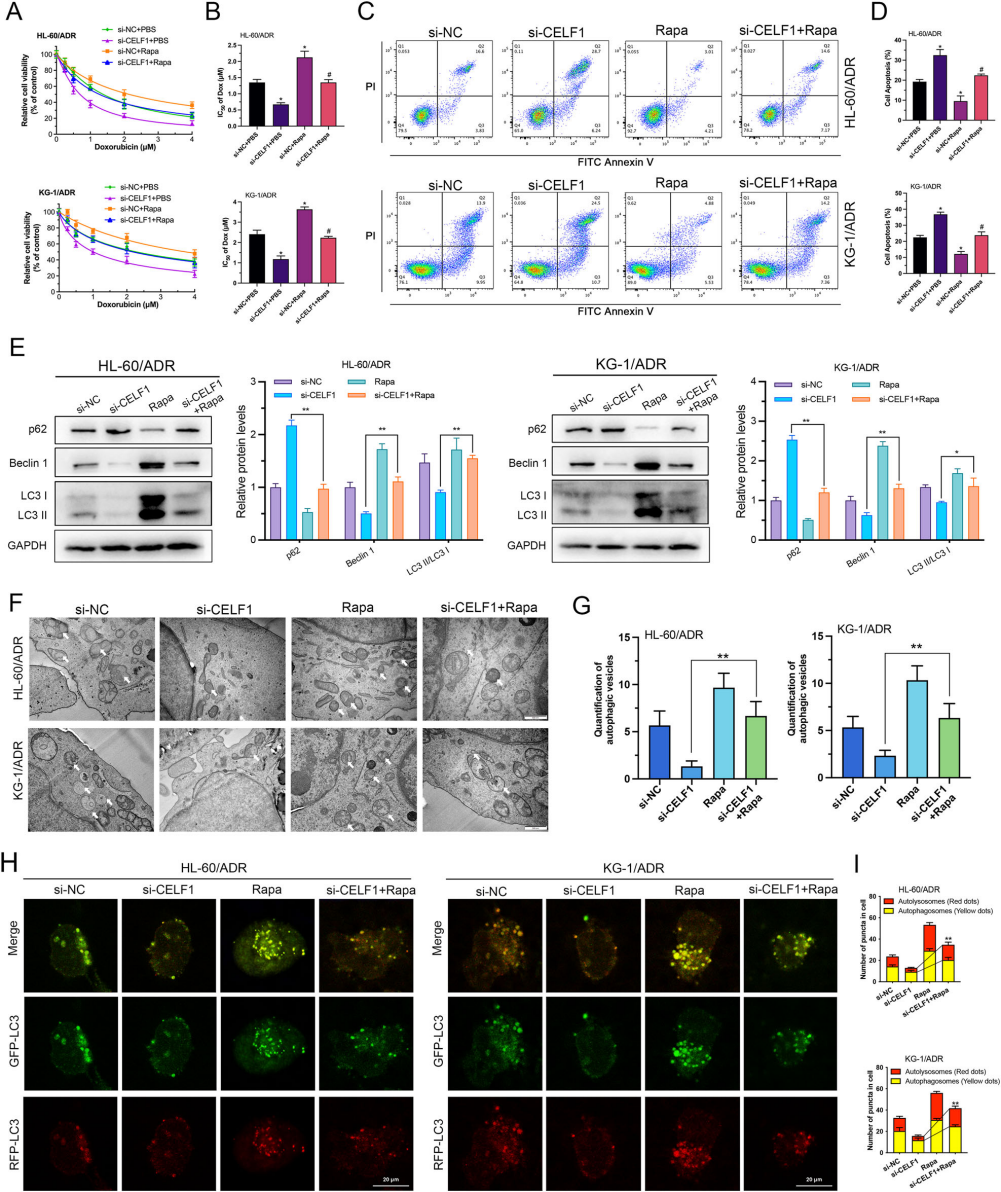
ADR resistance and autophagy induction following CELF1 knockdown and Rapa treatment. **A**, **B** CCK8 assess IC_50_ values and inhibition curves after CELF1 knockdown and Rapa treatment in AML cells. **C**, **D** Flow cytometry analysis of apoptosis in cells following CELF1 knockdown and Rapa treatment. **E** Western blot analysis of autophagy markers in cells after CELF1 knockdown and Rapa treatment. **F**, **G** Autophagic organelles visualized by transmission electron microscopy in cells after CELF1 knockdown and Rapa treatment. **H**, **I** Tandem mRFP-GFP-LC3 fluorescence assay performed in cells after CELF1 knockdown and Rapa treatment to monitor autophagic flux.

Previous studies have indicated that autophagy is a potential mechanism of resistance to various cytotoxic drugs in a range of cancers, including hematologic malignancies. Therefore, we further investigated whether CELF1 promotes ADR resistance in AML cells by inhibiting cellular autophagy. We conducted rescue experiments by treating KG-1/ADR and HL-60/ADR cells with the autophagy inducer rapamycin (Rapa). CCK-8 assay revealed that cells treated with Rapa exhibited higher ADR IC_50_ values compared to the si-NC group, indicating increased ADR resistance. This suggests that enhancing autophagy in AML cells indeed contributes to ADR resistance. Our previous findings demonstrated that depletion of CELF1 significantly reduced ADR resistance, and adding Rapa on this basis partially restored ADR resistance (Fig. 3A, B). Similarly, flow cytometry results showed that Rapa alone could inhibit cell apoptosis, while adding Rapa to CELF1 knockdown cells partially suppressed the apoptosis induced by CELF1 downregulation (Fig. 3C, D). This suggests that CELF1 likely promotes AML cell resistance to ADR through regulating cellular autophagy. Subsequently, we used Western blot analysis to examine the expression of autophagy markers. The results showed that compared to the si-NC group, p62 expression was significantly reduced in the Rapa group, while the ratio of LC3-II/LC3-I and Beclin-1 expression markedly increased, indicating that Rapa significantly enhanced cellular autophagy levels. In CELF1-knocked down cells treated with Rapa, p62 expression increased, and the ratio of LC3-II/LC3-I and Beclin-1 expression decreased significantly compared to the group with only CELF1 knockdown (Fig. 3E). Additionally, transmission electron microscopy results demonstrated an increase in autophagosome formation with Rapa treatment, with more autophagosomes observed in CELF1 knockdown cells treated with Rapa compared to the group with only CELF1 downregulation(Fig. 3F, G). The results of the tandem mRFP-GFP-LC3 fluorescence assay showed that treatment with Rapa alone significantly increased autophagic flux in HL-60/ADR and KG-1/ADR cells. However, knockdown of CELF1 expression in these cells partially reversed the Rapa-induced enhancement of autophagic flux(Fig. 3H, I). These suggest that the addition of Rapa can restore cellular autophagy and ADR drug sensitivity in CELF1 knockdown cells.

### ATG5 is a potential downstream target of CELF1

**Fig. 4:**
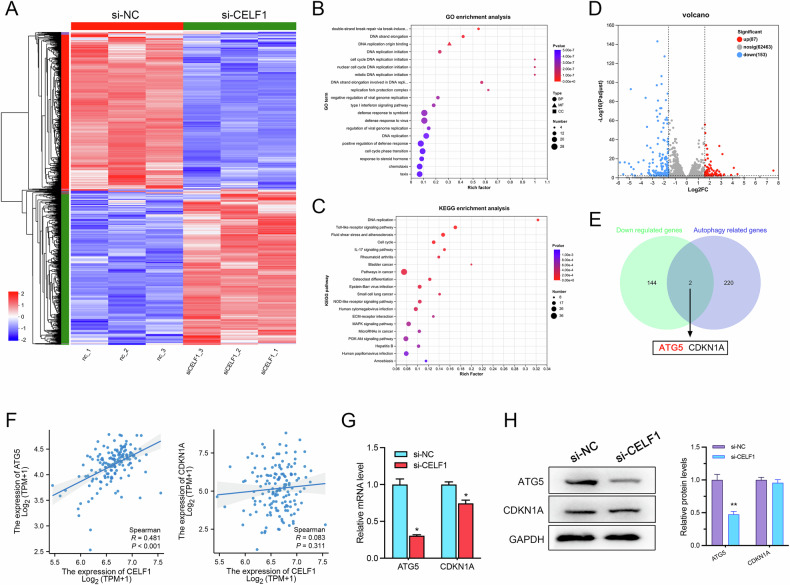
RNA-seq analysis of potential downstream targets following CELF1 depletion. **A** The heat map illustrates DEGs following CELF1 depletion. **B** The top 20 enriched GO terms among DEGs. **C** The top 20 enriched KEGG pathways among DEGs. **D** Volcano plot showing 240 DEGs following CELF1 knockdown. **E** Venn diagram displays the common genes between the downregulated coding genes and the HADb. **F** Spearman’s correlation analysis of ATG5, CDKN1A with CELF1 expression in the AML profiles of TCGA dataset. **G**, **H** RT-qPCR and Western blot analysis the expression of ATG5 and CDKN1A following CELF1 depletion.

In order to identify downstream target genes regulated by CELF1 in AML cells, we performed RNA-seq high-throughput sequencing on KG-1/ADR cells before and after CELF1 knockdown. The transcriptome analysis encompassed 33,094 genes, of which 987 showed statistically significant changes in expression following CELF1 knockdown, with 490 genes upregulated and 497 genes downregulated (|log2FC|≥ 1, *P* < 0.05). The heatmap displayed illustrates the top 100 upregulated and downregulated genes (Fig. 4A). In the GO enrichment analysis, several GO terms related to DNA replication and cell cycle processes were enriched, including DNA replication initiation, DNA strand elongation, double-strand break repair, and cell cycle phase transition, which were the most enriched biological processes. This finding was consistent with our cell proliferation data (Fig. 4B). In the KEGG pathway enrichment analysis (Fig. 4C), the most significant pathways, listed in ascending order of *p*-value, included DNA replication, Cell cycle, Pathways in cancer, MAPK signaling pathway, and PI3K-Akt signaling pathway. To identify CELF1 target genes, we applied more stringent criteria to the differentially expressed genes from sequencing, using a *P* < 0.01 and a fold change of 3, presented in a volcano plot (Fig. 4D). After excluding non-coding genes from the 153 downregulated genes, we obtained 146 protein-coding genes. Intersection analysis with the Human Autophagy Database(HADb) revealed ATG5 and CDKN1A as potential target genes of CELF1 (Fig. 4E). Furthermore, analysis of the correlation between CELF1 and ATG5/CDKN1A expression in the TCGA database showed a significant positive correlation between CELF1 and ATG5 (R = 0.481, *P* < 0.001), while no significant correlation was found between CELF1 and CDKN1A (R = 0.083, *P* = 0.311) (Fig. 4F). Subsequent RT-qPCR and Western blot analyses demonstrated that knocking down CELF1 significantly reduced ATG5 expression, while the expression of CDKN1A did not show significant changes (Fig. 4G, H), indicating that ATG5 is an important target gene regulated by CELF1 in controlling cellular autophagy.

### CELF1 interacts with ATG5 mRNA and maintains its stability

**Fig. 5:**
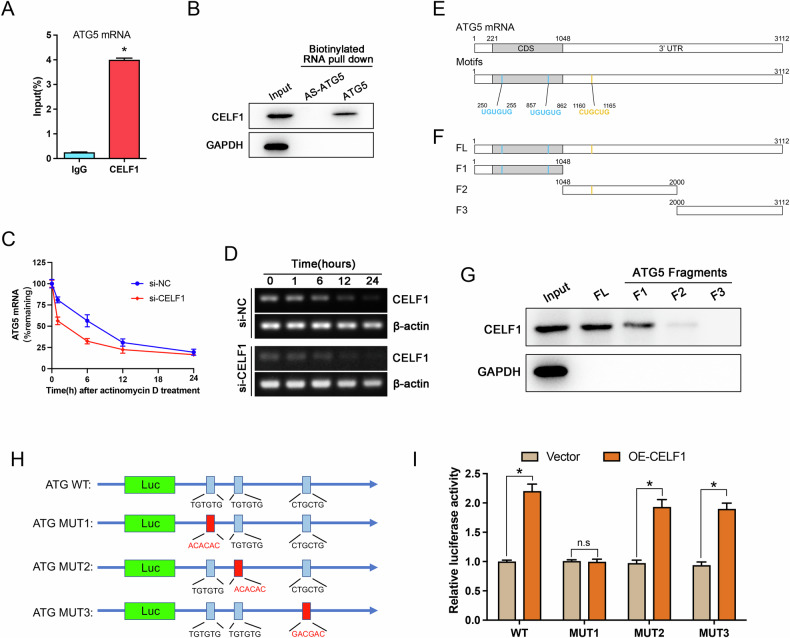
CELF1 binds to ATG5 mRNA and preserves its stability. **A** RIP-qPCR analysis confirmed CELF1 binding to ATG5 mRNA, normalized against the input group with IgG as the negative control. **B** The RNA pull-down assay and western blot analysis confirmed the direct binding of biotinylated ATG5 to CELF1 proteins. **C**, **D** CELF1 depletion was studied for its effect on ATG5 mRNA stability in AML cells by actinomycin D-chase experiments. **E**, **F** Schematic representation of CELF1 binding motifs on ATG5 and the fragmented ATG5 mRNA biotinylated transcripts. **G** Representative CELF1 immunoblots of the material pulled down by the different biotinylated fragments of the ATG5 mRNA. **H** Binding sites of CELF1 on ATG5 and mutant sequences. **I** Dual-luciferase reporter assay demonstrating CELF1-mediated regulation of ATG5 mRNA abundance.

To further explore the potential molecular mechanism of CELF1 in regulating ATG5 expression, we conducted RIP assays to validate the targeting of ATG5 mRNA by CELF1 protein. The combined results of RIP and RT-qPCR showed that the CELF1-specific antibody effectively co-precipitated ATG5 mRNA compared to the negative IgG control (Fig. 5A). Additionally, using biotin-labeled ATG5 mRNA sequences and their reverse sequences in RNA pull-down experiments, Western blot results demonstrated specific interaction between ATG5 mRNA and CELF1 protein, confirming their relationship (Fig. 5B). In mRNA decay experiments, after actinomycin D treatment, the abundance of ATG5 mRNA in the CELF1 knockdown group decreased significantly over time compared to the control group, indicating that CELF1 specifically binds to ATG5 mRNA in AML cells and increases its half-life (Fig. 5C, D).

Previous studies have shown that CELF1, as a crucial RNA-binding protein, can specifically recognize CUG, UG, and UA repeat sequences (such as CUGCUG, UGUGUG, UAUAUA, etc.) [25–27]. Through sequence alignment, we identified two UGUGUG sites in the CDS region and one CUGCUG site in the 3’UTR region of ATG5 mRNA (Fig. 5E). Subsequently, we fragmented ATG5 mRNA into smaller transcripts to determine specific binding sites for CELF1. By constructing biotin-labeled full-length ATG5 transcript (FL) and three region fragments (F1, F2, and F3), we conducted RNA pull-down analysis (Fig. 5F). The results showed the strongest binding affinity of CELF1 to the F1 region, weaker binding to the F2 region, and no observed binding to F3 (Fig. 5G). These findings clearly demonstrate that CELF1 can specifically bind to ATG5 mRNA, with its highest affinity spanning the CDS region.

To elucidate whether CELF1 regulates the stability of ATG5 mRNA by specifically recognizing the UGUGUG motif and to demonstrate the efficiency and effect of the binding site, we designed mutated sequences for the binding site and created a model diagram (Fig. 5H). Subsequently, we constructed luciferase reporter vectors containing either the wild-type ATG5 mRNA (with the UGUGUG motif) or the mutated sequence (ACACAC) and transfected them into 293T cells. By co-transfecting with the OE-CELF1 plasmid and measuring changes in luciferase activity, we found that CELF1 overexpression significantly increased luciferase activity in the wild-type group, indicating an enhancement in mRNA stability or translation efficiency, while this effect was completely abolished in the MUT1 (_250_UGUGUG_255_) (Fig. 5H). These results provide direct evidence that CELF1 regulates the stability of ATG5 mRNA by binding to its UGUGUG motif, offering molecular-level insights into their functional interaction.

### CELF1 regulates autophagy in AML cells via ATG5 to promote ADR resistance

**Fig. 6:**
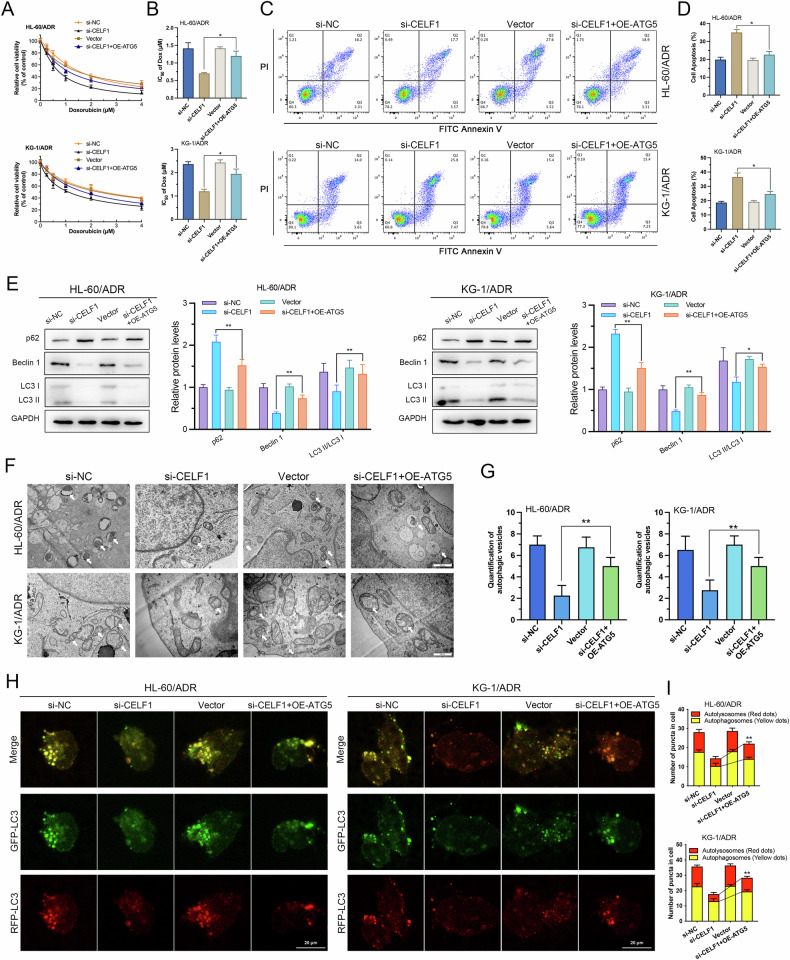
CELF1 modulates AML cell autophagy via ATG5 to combat ADR resistance. **A**, **B** CCK8 assess IC_50_ values and inhibition curves after CELF1 knockdown and ATG5 upregulation in AML cells. **C**, **D** Flow cytometry analysis of apoptosis in cells following CELF1 knockdown and ATG5 upregulation. **E** Western blot analysis of autophagy markers in cells after CELF1 knockdown and ATG5 upregulation. **F**, **G** Autophagic organelles visualized by transmission electron microscopy in cells after CELF1 knockdown and ATG5 upregulation. **H**, **I** Tandem mRFP-GFP-LC3 fluorescence assay performed in cells after CELF1 knockdown and ATG5 upregulated to monitor autophagic flux.

To confirm whether CELF1 influences cellular autophagy and ADR resistance by targeting ATG5, we conducted rescue experiments, with the overexpression efficiency of ATG5 demonstrated in Supplementary Fig. S2C, D. The results from the CCK-8 assay showed that Compared to the si-NC group, the IC_50_ of the si-CELF1 group was significantly reduced. Furthermore, following the knockdown of CELF1, overexpression of ATG5 resulted in a significant increase in the IC_50_ value in the si-CELF1 + OE-ATG5 group compared to the si-CELF1 group, indicating that overexpression of ATG5 partially reversed the increased sensitivity to ADR in CELF1-knocked down KG-1/ADR and HL-60/ADR cells (Fig. 6A, B). Similarly, flow cytometry results demonstrated that in KG-1/ADR and HL-60/ADR cells with CELF1 knockdown alone, apoptosis was significantly higher compared to the control group, while in the rescue group with ATG5 overexpression on the basis of CELF1 knockdown, this phenomenon was significantly reversed (Fig. 6C, D). This suggests that CELF1 likely mediates ADR resistance through targeting ATG5. Furthermore, we used Western blot analysis to examine autophagy-related proteins. The results showed that overexpression of ATG5 reversed the decreased ratio of LC3-II/LC3-I and Beclin-1 levels, and reduced the upregulation of p62 in CELF1-knocked down KG-1/ADR and HL-60/ADR cells (Fig. 6E). Additionally, transmission electron microscopy results indicated that knocking down CELF1 reduced the formation of autophagosomes in cells, while a slight increase in the number of autophagosomes was observed in cells overexpressing ATG5 on the basis of CELF1 knockdown (Fig. 6F, G). The tandem mRFP-GFP-LC3 reporter assay demonstrated that CELF1 knockdown significantly suppressed cellular autophagic flux. Notably, this suppression was partially reversed by ATG5 overexpression in CELF1- depleted cells (Fig. 6H, I). This suggests that CELF1 can regulate cellular autophagy through ATG5.

In addition, we explored the effect of Rapa on the mTOR pathway in drug-resistant cells to determine whether the resistance and the effect of rapamycin arise from different pathways. Rapa treatment significantly reduced phosphorylated mTOR (p-mTOR) levels in drug-resistant cells while markedly inducing autophagy (Supplementary Fig. S3A). These findings indicate that Rapa effectively inhibits mTOR activity in drug-resistant cells, thereby relieving its suppression on autophagy. Further investigation demonstrated that CELF1 knockdown did not alter the expression of mTOR pathway-related proteins (Supplementary Fig. S3B), suggesting that the drug resistance mechanism regulates autophagy through the CELF1-ATG5 axis independently of the mTOR pathway. This evidence further supports the critical regulatory role of the CELF1-ATG5 axis in AML drug resistance.

### CELF1 knockdown combined with ADR treatment improves AML xenograft model survival

**Fig. 7:**
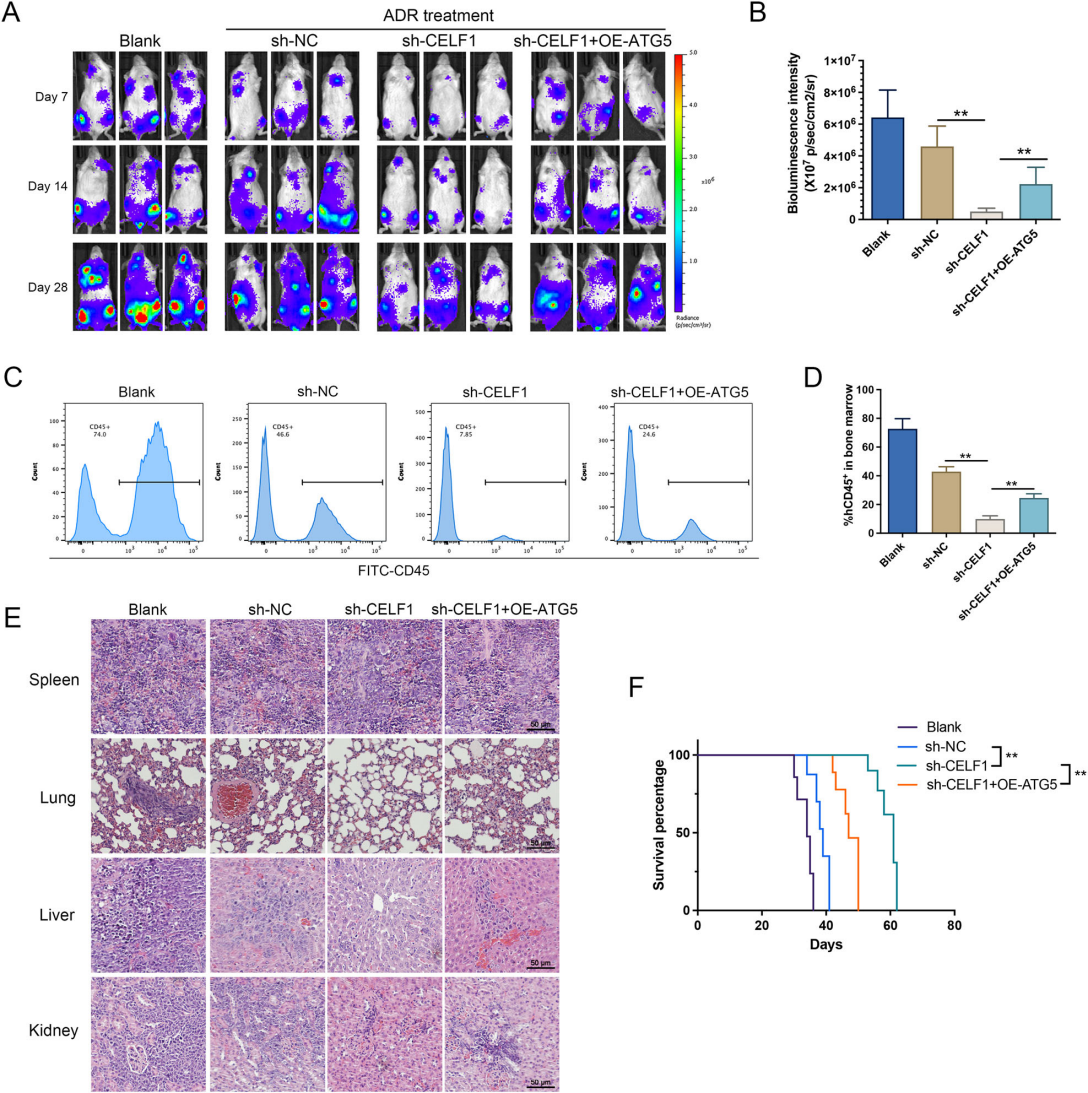
CELF1 depletion combined with ADR treatment reduced leukemia progression in an AML mouse model. **A**, **B** Comparison of the evolution of bioluminescence emission in mice treated with ADR in combination with each group or the untreated group after injection of HL-60/ADR Luci cells, measured by the IVIS Spectrum. **C**, **D** CD45^+^ cell proportion in mouse bone marrow assessed by flow cytometry in each group. **E** H&E staining shows that CELF1 knockdown reduces leukemic infiltration and preserves tissue structure in the spleen, lung, liver and kidney, while ATG5 overexpression partially reverses this effect. **F** Kaplan–Meier survival curves of the four different treatment groups.

To further elucidate the role of CELF1 in AML cell resistance in vivo, we established a disseminated AML mouse model using HL-60/ADR cells stably expressing luciferase. Three days after cell injection, ADR treatment was administered, and bioluminescence imaging was performed on days 7, 14, and 28 to track the fate of the target cells. The Blank group received no drug treatment, and the In Vivo Imaging system revealed rapid growth of AML cells. The remaining three groups underwent ADR treatment under the same conditions. In vivo fluorescence imaging showed that stable knockdown of CELF1 significantly reduced fluorescence intensity compared to the NC group, indicating that CELF1 knockdown effectively increased tumor cell killing and sensitivity to ADR. Overexpression of ATG5 on the basis of CELF1 knockdown increased fluorescence intensity, suggesting that the rescue effect of ATG5 partially reversed cell ADR resistance (Fig. 7A, B). Furthermore, bone marrow cells from some mice after 28 days were subjected to flow cytometry analysis. The results showed that the Blank group had a high positivity rate of CD45^+^ cells at 74%, while the NC group had 46.6% CD45^+^ cells. In the CELF1 knockdown group, the CD45^+^ cell proportion was only 7.85%, significantly lower than the NC group, and in the ATG5 rescue group, the CD45^+^ cell proportion recovered to 24.6% (Fig. 7C, D). Histological analysis of the spleen, lung, liver, and kidney tissue revealed differential levels of infiltration and tissue damage among the groups. The Blank and NC groups displayed severe tissue infiltration and structural damage, while the CELF1 knockdown group exhibited significantly reduced infiltration and preservation of tissue architecture. In contrast, the ATG5 rescue partially reversed these protective effects, as evidenced by increased infiltration and damage in comparison to the CELF1 knockdown group (Fig. 7E). Additionally, all ADR treatment groups showed extended survival, with the CELF1 knockdown combined treatment group exhibiting the best survival, while ATG5 rescue weakened some of the treatment effects(Fig. 7F). It is evident that knockdown CELF1 expression effectively enhances the therapeutic effect of ADR on AML xenografts, and this effect is mediated through the regulation of ATG5 expression.

## Discussion

Acute Myeloid Leukemia (AML), a severe malignant hematologic tumor, originates from hematopoietic stem cell progenitors [2]. Among the eight subtypes of AML (M0-M7), only Acute Promyelocytic Leukemia (APL, M3 subtype) can achieve clinical remission rates of over 95% with the use of cytarabine (Ara-C) or all-trans retinoic acid (ATRA) [28, 29]. However, other types of AML, while controllable with standard chemotherapy, lead to relapse or drug resistance in around 40% of young patients and the majority of elderly patients [30]. The issue of chemoresistance in AML poses a critical challenge in clinical treatment. Numerous studies suggest that RNA-binding proteins (RBPs) play crucial regulatory roles in cells, participating in processes such as gene transcription, splicing, and translation. Aberrant expression of RBPs in tumor cells can alter cellular responses to chemotherapy [31]. For instance, abnormal RBP expression can affect the expression or regulation of drug target genes, leading to reduced sensitivity of tumor cells to drugs [32]. It may also disrupt normal cellular signaling pathways, promoting cell proliferation or inhibiting apoptosis, thereby enhancing drug resistance [33, 34]. Therefore, there is an urgent need to broaden our understanding of the relationship between RBPs and AML drug resistance, identify new therapeutic targets for AML clinical resistance, and provide a new theoretical basis for treating relapsed AML.

CELF1, also known as CUG-repeat binding protein 1 (CUGBP1), is a novel RNA-binding protein containing three RNA recognition motifs (RRMs) that bind to specific mRNAs and determine their fate [7, 27]. Studies have shown that CELF1 is primarily found in skeletal muscle, heart, and brain, initially discovered to be involved in the selective translation of muscle atrophy kinase in myotonic dystrophy [8, 35, 36]. Additionally, CELF1 plays important roles in the occurrence and development of various tumors. For example, CELF1 is overexpressed in gastric and lung cancers, and knocking down CELF1 significantly inhibits tumor cell proliferation [37, 38]. In gliomas, CELF1 is upregulated and promotes cell proliferation by inhibiting CDKN1B [39]. Furthermore, CELF1 enhances cell migration, invasion, and chemoresistance in colorectal cancer by targeting ETS2 [10]. In this study, analysis of TCGA data revealed significantly higher expression of CELF1 in AML patients compared to normal individuals, suggesting a potential important role for CELF1 in AML progression. To further investigate the relationship between CELF1 expression levels in AML cells and ADR resistance, we established two AML cell lines with acquired resistance, analyzed the differential expression of CELF1 between these cells and their parental cell lines, and found significantly elevated expression of CELF1 in the drug-resistant AML cells. Using CCK-8 assays and siRNA knockdown techniques, we identified CELF1 as a key molecule involved in acquired AML drug resistance. Additionally, we found that knocking down CELF1 affects cellular autophagy levels, which is a critical factor in tumor drug resistance. Therefore, in-depth exploration of CELF1’s regulation of autophagy-mediated AML drug resistance holds significant clinical significance.

Cell autophagy is a fundamental physiological process through which cells self-digest damaged or excessive cellular components and structures to maintain intracellular homeostasis, energy balance, and promote cell renewal and remodeling [40]. Under normal conditions, autophagy plays a crucial role in cell health and longevity. However, in certain abnormal states such as disease or aging, autophagy may become dysregulated, leading to cellular abnormalities [41, 42]. Previous studies have shown that cellular autophagy can be a survival mechanism triggered by stress conditions like cancer treatment, often contributing to chemotherapy resistance and long-term dormancy of tumor cells, ultimately leading to advanced clinical symptoms of relapse and metastasis [43]. For example, docetaxel treatment of triple-negative breast cancer induces autophagy, inactivating the NF-κB signaling pathway, reducing apoptosis rates, and increasing resistance to Epirubicin [44]. Autophagy is involved in cadmium resistance in lung epithelial fibroblasts by inducing multidrug resistance protein 1 (MRP1) and balancing endoplasmic reticulum stress [45]. Additionally, Docetaxel promotes the expression of high mobility group box 1 (HMGB1), enhancing the formation of the autophagy core complex Beclin-1-PI3K-III through the MEK/ERK1/2 pathway, leading to resistance to Docetaxel [46]. Currently, several chemotherapy drugs used in AML treatment, such as ADR and cytarabine, can induce autophagy as a survival mechanism to resist cytotoxic stress and counteract the therapeutic effects of these drugs [47]. Therefore, identifying the role and potential mechanisms of CELF1 in autophagy regulation may be an effective strategy to enhance efficacy and overcome resistance in AML treatment.

To uncover the potential mechanisms by which CELF1 drives acquired drug resistance in AML cells, we conducted RNA-seq high-throughput sequencing analysis of KG-1/ADR cells before and after CELF1 knockdown, revealing significant changes in 987 genes upon CELF1 knockdown. Many of these genes were enriched in Gene Ontology terms related to DNA replication and the cell cycle. In the KEGG pathway enrichment analysis, pathways such as DNA replication, the cell cycle, pathways in cancer, the MAPK signaling pathway, and the PI3K-Akt signaling pathway were enriched. This suggests that CELF1 likely promotes AML cell resistance by regulating downstream target genes involved in these pathways. Subsequently, we identified two downstream target genes of CELF1 involved in regulating autophagy, ATG5 and CDKN1A, through analysis with the Human Autophagy Database. Expression correlation analysis revealed a strong positive correlation between CELF1 and ATG5, while no significant correlation was found between CELF1 and CDKN1A. Our RT-qPCR and Western blot results showed significant downregulation of ATG5 and no significant change in CDKN1A expression upon CELF1 knockdown. Rescue experiments both in vitro and in vivo further confirmed that CELF1 enhances AML cell tolerance to ADR, with CELF1 increasing cell resistance through regulation of ATG5 expression. Autophagy is a tightly regulated process, with key participants being autophagy-related (ATG) proteins. Among the identified 41 ATG genes, ATG5 is essential for autophagosome formation and positively influences autophagic activity [48]. m^6^A-modified USP13 induces autophagy and imatinib resistance in gastrointestinal stromal tumor cells by regulating ATG5 stability [49]. miR-137 promotes sensitivity of pancreatic cancer cells to ADR by inhibiting ATG5-mediated autophagy [50]. Additionally, exosomal LOC85009 regulates cell autophagy to inhibit resistance to docetaxel through the USP5/USF1/ATG5 axis [51]. Therefore, ATG5 serves as a crucial intermediate link in CELF1-regulated autophagy and ADR resistance in AML.

Subsequently, we performed RIP-qPCR and RNA pull-down experiments to confirm the direct interaction between CELF1 protein and ATG5 mRNA. Moreover, mRNA decay experiments demonstrated that knocking down CELF1 expression significantly reduced the half-life of ATG5 mRNA. Previous studies have shown that CELF1, also known as CUG-repeat binding protein 1 (CUGBP1), contains three RNA recognition motifs (RRMs) that specifically bind to CUG repeat sequences (such as CUGCUG) in the N-terminal RRM (RRM1 and RRM2), and UG repeat sequences (such as UGUGUG, CGUGUG) in the C-terminal RRM3, mediating alternative splicing events and mRNA stability [8, 25–27, 52]. Through sequence alignment, we found two UGUGUG sites in the CDS region and one CUGCUG site in the 3’UTR region of ATG5 mRNA. Binding activity experiments with different segments of CELF1 and ATG5 transcripts revealed significant differences in binding affinity between segments. Regions containing two UGUGUG sites showed the strongest binding capacity, while regions containing one CUGCUG site had weaker but synergistic binding capabilities. Therefore, CELF1 likely positively regulates ATG5 mRNA stability through specific binding to these repeat sequences, although the exact mechanism by which CELF1 promotes ATG5 mRNA stability remains to be elucidated.

In conclusion, this study demonstrates that CELF1 is a novel AML chemotherapy resistance-related protein that directly targets and regulates ATG5 mRNA stability, promoting its expression and triggering protective autophagy-mediated drug resistance in cells. Our findings provide new insights into the potential mechanisms of AML chemotherapy resistance, which will help clarify the precise regulatory mechanisms of AML chemotherapy resistance and offer a new perspective on CELF1 and other RBPs as novel therapeutic targets for AML.

## Materials and methods

### Bioinformatics analysis

The pan-cancer analysis of the CELF1 gene was conducted using data from The Cancer Genome Atlas (TCGA) database. CELF1 expression and clinical data for AML were acquired from the TCGA database. Preprocessing steps were performed, including normalization and filtering of low-expressed genes. To examine the correlation between CELF1 expression and ATG5 and CDKN1A in TCGA, Spearman correlation analysis was performed on log-transformed and normalized RNA-Seq data. Autophagy genes from the Human Autophagy Database (http://www.autophagy.lu/) were used for screening downstream target genes of CELF1. Analyzed data are available in Supplementary Table S1.

### Cell culture

The AML cell lines (HL-60, MOLM-13, U937, THP-1, and KG-1) and the human bone marrow stromal cell line (HS-5) were obtained from ATCC and cultured in appropriate growth media. The AML cells were maintained in RPMI-1640 medium supplemented with 10% FBS and 1% penicillin-streptomycin, while the HS-5 cells were cultured in DMEM supplemented with 10% FBS and 1% penicillin-streptomycin. Media were changed regularly to sustain optimal cell growth. All cells were incubated at 37 °C with 5% CO_2_ in a humidified atmosphere.

ADR-resistant cell lines were established using a stepwise selection method. The parental cell lines (HL-60 and KG-1) were gradually exposed to increasing concentrations of ADR over several months. Starting with a low concentration, the cells were cultured and passaged in progressively higher ADR concentrations until an ADR-resistant cell line was obtained.

### Transfection of cells

Cells were transfected with either siRNA targeting CELF1 to achieve knockdown or a ATG5 overexpression plasmid using Lipofectamine 2000. the specific siRNA and a negative control or overexpression plasmid and an empty vector control were diluted in serum-free growth medium and added to cells at 50–70% confluency. Following incubation, the cells were cultured at 37 °C for the appropriate duration to facilitate efficient siRNA and DNA uptake.

### Cell viability assay

Cell viability was assessed using the Cell Counting Kit-8 (CCK-8) assay according to the manufacturer’s instructions. Briefly, cells in each group were seeded into 96-well plates at a density of 2 × 10^3^ cells/well. After incubation for 48 h, the absorbance was determined by using a microplate reader set at a wavelength of 450 nm. The experiments were repeated in triplicate, independently.

### Cell apoptosis assay

Cell apoptosis was assessed using the Annexin V-FITC/PI staining assay followed by flow cytometry analysis. Briefly, cells subjected to experimental treatments were collected, washed with PBS, and stained with Annexin V-FITC and PI according to the assay kit instructions. Flow cytometry analysis was then performed to determine the percentage of apoptotic cells based on Annexin V-FITC and PI fluorescence signals. Statistical analysis was conducted to compare the percentage of apoptotic cells between experimental and control groups, with a significance level set at *P* < 0.05.

### RT-qPCR

TRIzol reagent was utilized to extract total RNA from cells. The concentration and purity of the extracted RNAs were assessed using a NanoDrop One Spectrophotometer. Subsequently, cDNA synthesis was carried out following the manufacturer’s instructions provided with the PrimeScript RT reagent Kit. Real-time quantitative PCR (RT-qPCR) was performed utilizing the TB Green Premix Ex Taq kit. The primers used were as follows: CELF1 F: ATGGCACAGACGGCTATCAAGG, CELF1 R: CACAGATGCTGCGCTGATTTGC; ATG5 F: GCAGATGGACAGTTGCACACAC, ATG5 R: GAGGTGTTTCCAACATTGGCTCA; β-actin F: TGGCACCCAGCACAATGAA, β-actin R: CTAAGTCATAGTCCGCCTAGAAGCA. Relative gene expression was normalized to β-actin based on the 2^−ΔΔCt^ method.

### Western blot assays

Cells were lysed in RIPA buffer supplemented with protease and phosphatase inhibitors. The protein concentration was determined using a BCA protein assay kit. Equal amounts of protein samples were separated by SDS-PAGE and transferred onto PVDF membrane. After blocking non-specific binding, the membrane was probed overnight at 4 °C with primary antibodies(CELF1, CST #95084; Autophagy Antibody Sampler Kit, CST #4445; mTOR, CST #2983; p-mTOR, CST #5536; CDKN1A, CST #2947) specific to the target proteins. Following washing, the membrane was incubated with HRP-conjugated secondary antibodies, and protein bands were visualized using ECL detection reagents.

### Transmission electron microscopy

Autophagic structures were observed using electron microscopy. Cells were seeded onto culture dishes and treated accordingly. Following treatment, cells were fixed, dehydrated, and embedded in resin. Ultrathin sections were cut, stained, and mounted on copper grids. Autophagic structures, including autophagosomes and autolysosomes, were examined under a transmission electron microscope operated. Representative images were captured to visualize the observed structures associated with autophagy.

### Tandem mRFP-GFP-LC3 reporter assay

Cells were transfected with the mRFP-GFP-LC3 plasmid, followed by designated pharmacological treatments (BafA1, rapamycin) or genetic manipulations (siRNA-mediated CELF1 knockdown, ATG5 overexpression). After 24–48 h, cells were fixed with 4% paraformaldehyde, permeabilized with 0.1% Triton X-100, and imaged using a confocal microscope. Yellow (GFP^+^/mRFP^+^) and red (GFP^−^/mRFP^+^) puncta representing autophagosomes and autolysosomes, were counted in cells per group using ImageJ, respectively. Autophagic flux was calculated as the ratio of red-to-yellow puncta across experimental conditions.

### RNA sequencing

Total RNA was isolated from CELF1 knockdown or control HL-60/ADR cells by using Trizol reagent. The quality and quantity of the RNA samples were assessed with a spectrophotometer. The high‐throughput sequencing and analyses for mRNA were carried out by Majorbio Technology (Shanghai, China). Analyzed data are available in Supplementary Table S1.

### RIP-qPCR

Approximately 1 × 10^7^ HL-60/ADR cells were harvested and rinsed with PBS. RNA immunoprecipitation (RIP) was conducted using the Magna RIP Kit (Millipore) following the manufacturer’s instructions. In brief, cells were lysed for 60 min on ice in RIP Lysis Buffer supplemented with protease inhibitor and RNase inhibitor. Then, 50 μL of magnetic beads were coupled with either anti-CELF1 antibodies or anti-rabbit IgG antibodies. The cell lysates were incubated with the bead pellet overnight at 4 °C. Immunoprecipitated RNA was extracted from the pellet using phenol chloroform, while the immunoprecipitate underwent proteinase K treatment and subsequent RT-qPCR analysis.

### RNA pull-down assay

ATG5 or antisense RNA was transcribed in vitro from the pcDNA3.1 vector using the TranscriptAid T7 High Yield Transcription Kit. The transcribed ATG5 was purified with the PureLink RNA Mini Kit and subsequently biotin-labeled using the Biotin 3’ End DNA Labeling Kit. Next, the biotinylated RNA was incubated with whole-cell lysate from HL-60/ADR cells at room temperature for 1 h. The RNA-protein complex was then isolated using streptavidin magnetic beads, and the bound proteins were further extracted for subsequent Western blot analysis.

Three biotinylated RNA fragments spanning the ATG5 mRNA sequence were generated. Whole cell lysates from 293T cells overexpressing CELF1 were incubated with the biotinylated RNA fragments for 1 h at room temperature to allow for RNA-protein complex formation. Streptavidin magnetic beads were then used to isolate the RNA-protein complexes, and the bound proteins were extracted for subsequent Western blot analysis.

### RNA stability assay

To assess ATG5 mRNA stability, HL-60/ADR cells were treated with Actinomycin D, a transcriptional inhibitor. After seeding, Actinomycin D was added at 5 μM, while control cells received vehicle treatment. Cells were then incubated for various time intervals to allow for RNA degradation. Total RNA was extracted from harvested cells using a TRIzol and assessed for quality and quantity. Subsequently, cDNA synthesis was performed, and the expression levels of target RNAs were measured at each time point using quantitative real-time PCR or other appropriate techniques. Data were normalized to housekeeping genes and analyzed to determine the rate of RNA degradation under Actinomycin D treatment.

### CELF1-mediated drug resistance of AML cells in vivo

Immunocompromised NSG mice (5 weeks old) were purchased from Shanghai SLAC Laboratory Animal Co., Ltd. HL-60/ADR cells transfected with sh-CELF1 or OE-ATG5 resuspended in PBS were injected directly into via tail vein to mimic systemic dissemination. Tumor growth and progression were monitored by measuring leukemic burden in peripheral blood samples obtained at different time points. Fluorescence imaging was employed to visualize the localization and intensity of fluorescently labeled AML cells within live animals. All the mice were performed euthanasia after the experiment to ensure the welfare and ethical treatment of the animals involved.

### H&E staining

Tissues (spleen, lung, liver, and kidney) were collected, fixed in 4% paraformaldehyde, and embedded in paraffin. Sections (4 µm) were deparaffinized, rehydrated, and stained with hematoxylin for 5 min, followed by differentiation in 1% acid-ethanol and bluing in tap water. Slides were counterstained with eosin for 2 min, dehydrated, cleared in xylene, and mounted with neutral resin. Images were captured using a light microscope to assess tissue infiltration and structural integrity across experimental groups.

### Statistical analysis

All statistical analyses were performed using GraphPad Prism 10 software. Data were expressed as mean ± standard deviation (SD) or as indicated. A Student’s *t* test was used to analyze two group comparisons. Additionally, correlation analysis was conducted to evaluate the association between variables of interest. Differences were considered statistically significant at *P* < 0.05.

## Supplementary information

Table S1

Supplementary Figures and Figure legends

Original data files of Western blot

Original data files (qPCR original Ct values)

## Data Availability

The authors declare that all data supporting the findings of this study are available within the article.
