# Supplementary material for: The RNA-binding protein CELF1 targets ATG5 to regulate autophagy and promote drug resistance in acute myeloid leukemia
Source: Cell Death Dis. 2025 Aug 8;16(1):599. doi: 10.1038/s41419-025-07926-0 (PMC12334686; doi:10.1038/s41419-025-07926-0)
Supplement: Supplementary file 2 — Supplementary Figures and Figure legends [file 41419_2025_7926_MOESM2_ESM.docx]

**The RNA-binding protein CELF1 targets ATG5 to regulate autophagy and promote drug resistance in acute myeloid leukemia**

Xiaoyan Li^1^, Qiyi Qian^1^, Juejiashan Li^1^, Lu Zhang^2^, Lifang Wang^1^, Dongsheng Huang^3*^, Qiuran Xu^3*^, Wenhu Chen^1*^

^1^  School of Basic Medical Sciences & Forensic Medicine, Hangzhou Medical college, Hangzhou, 310053, China.

^2^  School of Life Sciences, Zhejiang Chinese Medical University, Hangzhou, 310053, China.

^3^  Zhejiang Key Laboratory of Tumor Molecular Diagnosis and Individualized Medicine, Zhejiang Provincial People's Hospital, Affiliated People's Hospital, Hangzhou Medical College, Hangzhou, 310014, Zhejiang, China.

**Correspondence：**Wenhu Chen (chenwenhu@hmc.edu.cn, Hangzhou medical college, No. 481 Binwen Road, 310053 Hangzhou, Zhejiang, P.R.China); Qiuran Xu (xuqiuran@hmc.edu.cn, Hangzhou medical college, No. 481 Binwen Road, 310053 Hangzhou, Zhejiang, P.R.China); Dongsheng Huang (dshuang@hmc.edu.cn, Hangzhou medical college, No. 481 Binwen Road, 310053 Hangzhou, Zhejiang, P.R.China).

**Supplementary figures and figure legends**

**
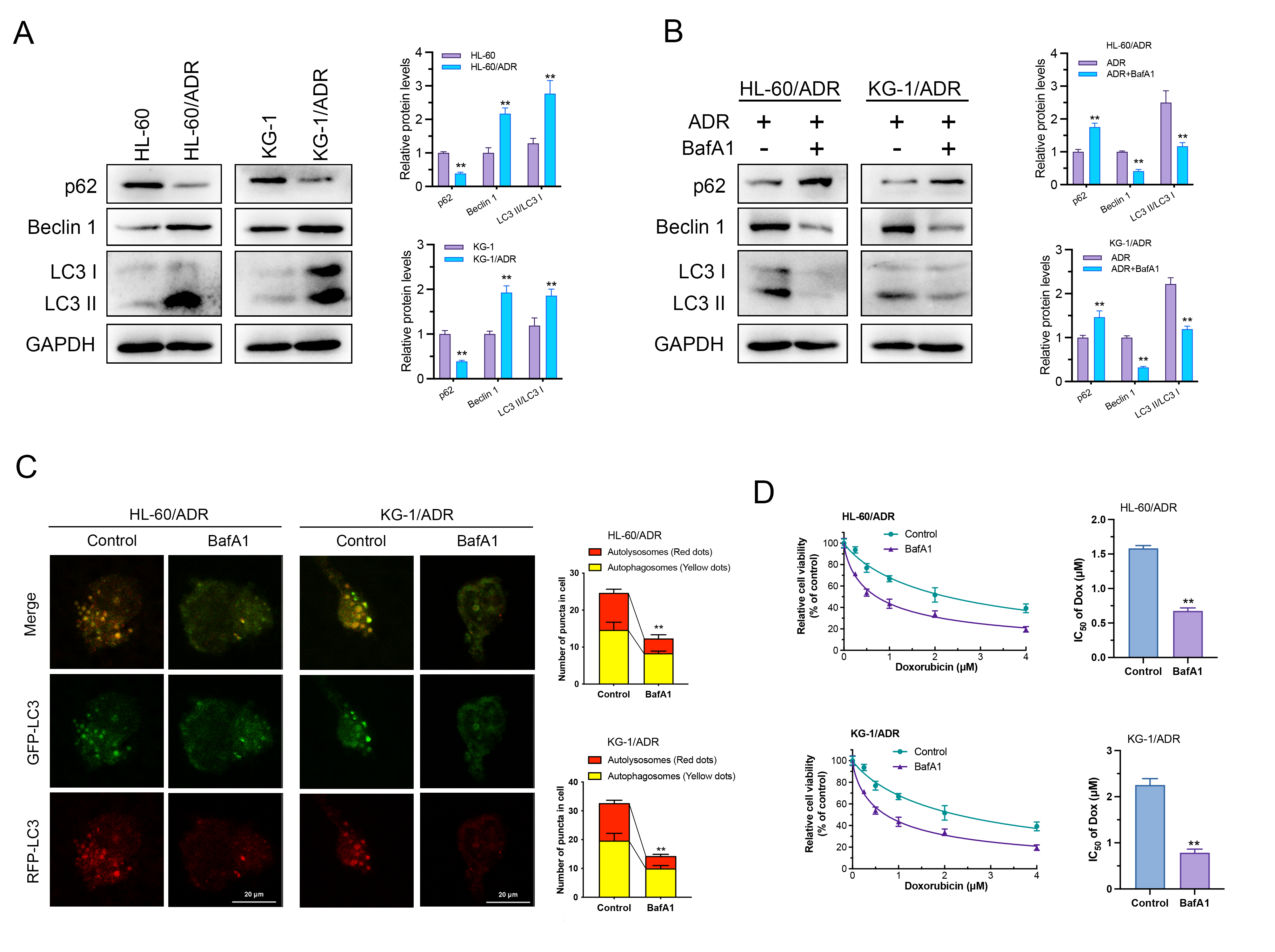
**

**Figure S1 Impact of autophagy intervention models on ADR resistance in AML cells**

(A) Western blot analysis comparing autophagy markers (p62, Beclin-1, LC3-II/LC3-I) between ADR-resistant (HL-60/ADR, KG-1/ADR) and parental (HL-60, KG-1) AML cells. (B) Impact of autophagy inhibition (BafA1) on autophagic activity in resistant cells, assessed via LC3-II/LC3-I and p62 levels. (C) Tandem GFP-mRFP-LC3 fluorescence assay to monitor autophagic flux (autophagosome-lysosome fusion) in resistant cells with or without BafA1 treatment. (D) CCK-8 assays evaluating ADR sensitivity in resistant cells following autophagy inhibition (BafA1).

**
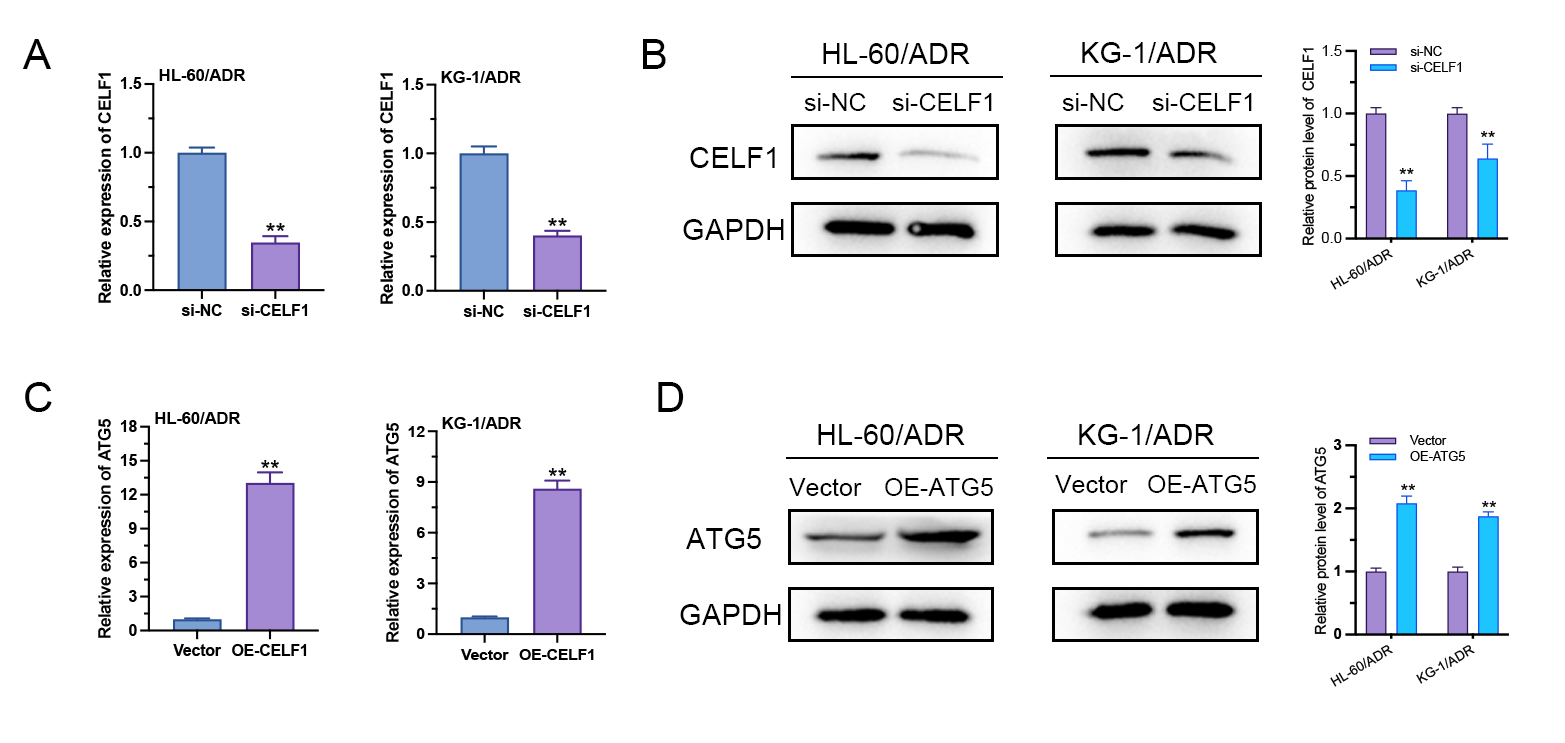
**

**Figure S2 Evaluation of CELF1 knockdown and ATG5 overexpression in AML cell lines**

(A, B) The efficiency of CELF1 knockdown in HL-60/ADR and KG-1/ADR cells was assessed using RT-qPCR and Western Blot analyses. (C, D) The efficiency of ATG5 overexpression in HL-60/ADR and KG-1/ADR cells was evaluated using RT-qPCR and Western Blot analyses.


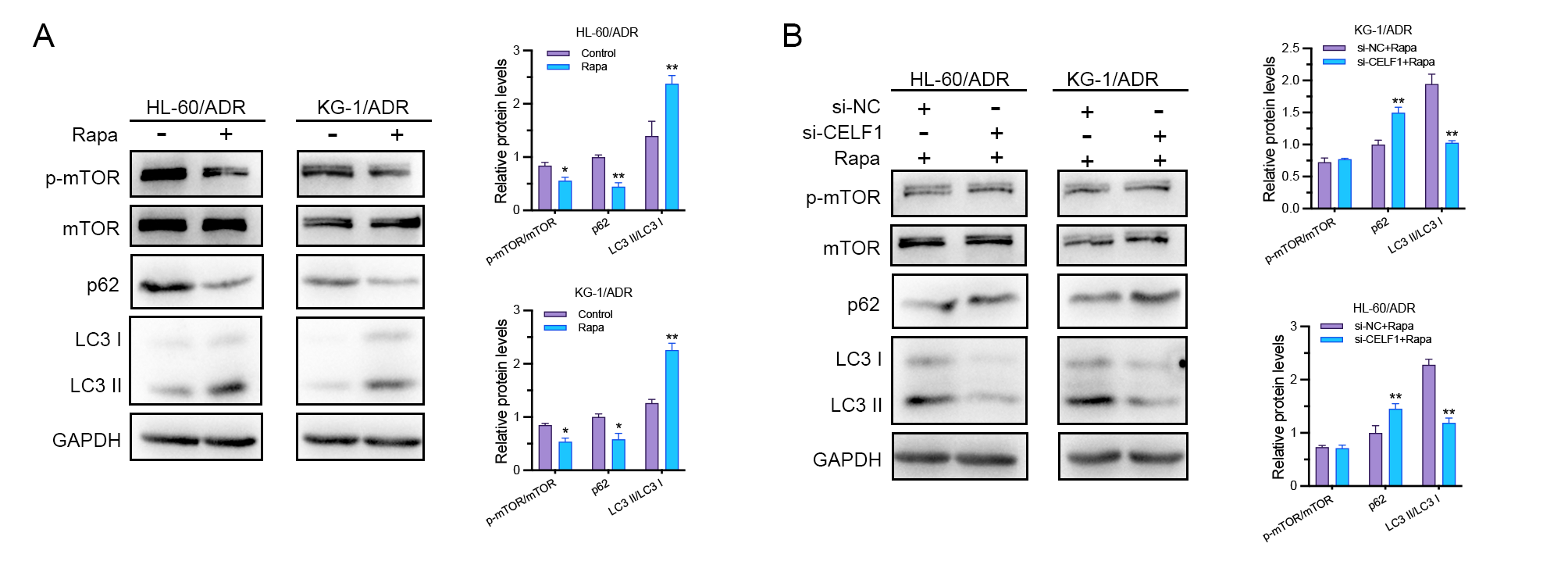


**Figure S3 mTOR pathway modulation and CELF1-ATG5 axis dependency in AML drug resistance**

(A) Western blot analysis of phosphorylated mTOR (p-mTOR) and autophagy markers in drug-resistant AML cells treated with rapamycin (Rapa) to assess mTOR pathway inhibition and autophagy induction. (B) Western blot evaluation of mTOR pathway-related proteins in CELF1-knockdown drug-resistant cells to determine mTOR signaling dependency.
