# Supplementary figures and images for: The RNA-binding protein CELF1 targets ATG5 to regulate autophagy and promote drug resistance in acute myeloid leukemia
Source: Cell Death Dis. 2025 Aug 8;16(1):599. doi: 10.1038/s41419-025-07926-0 (PMC12334686; doi:10.1038/s41419-025-07926-0)

Fig. 1

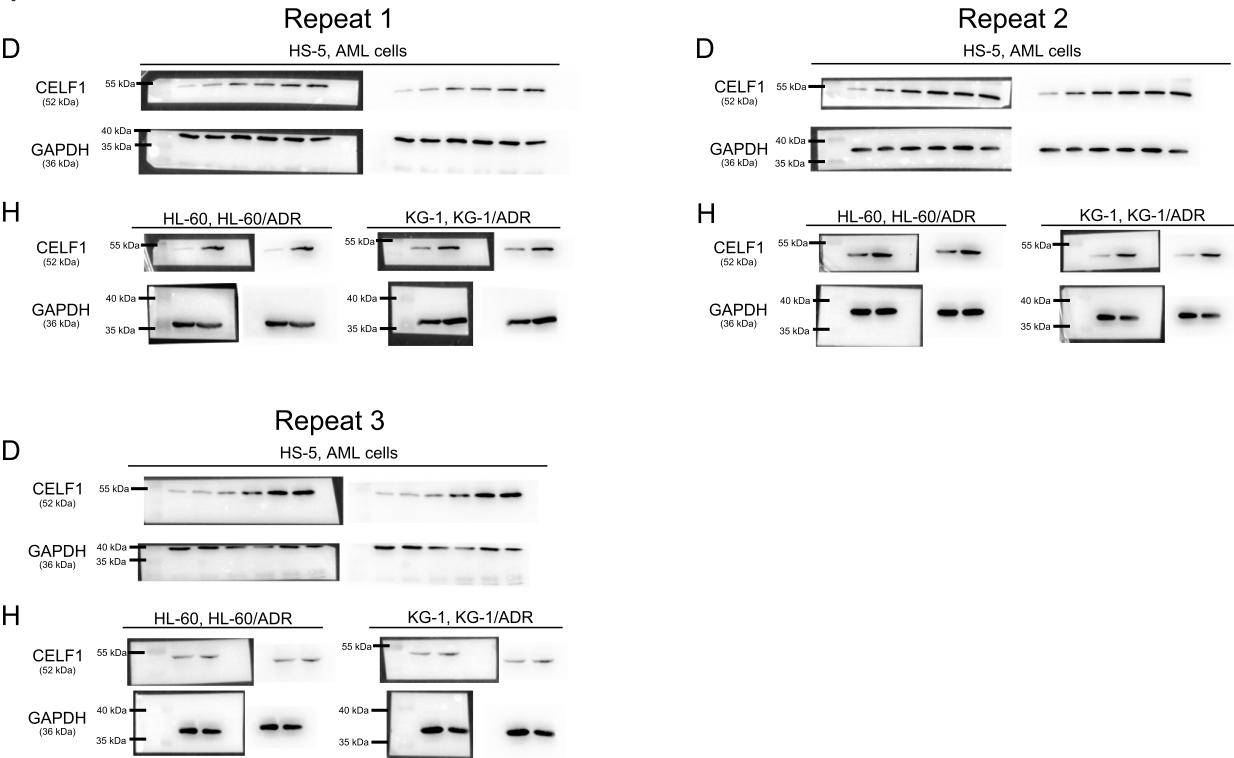

Fig. 2

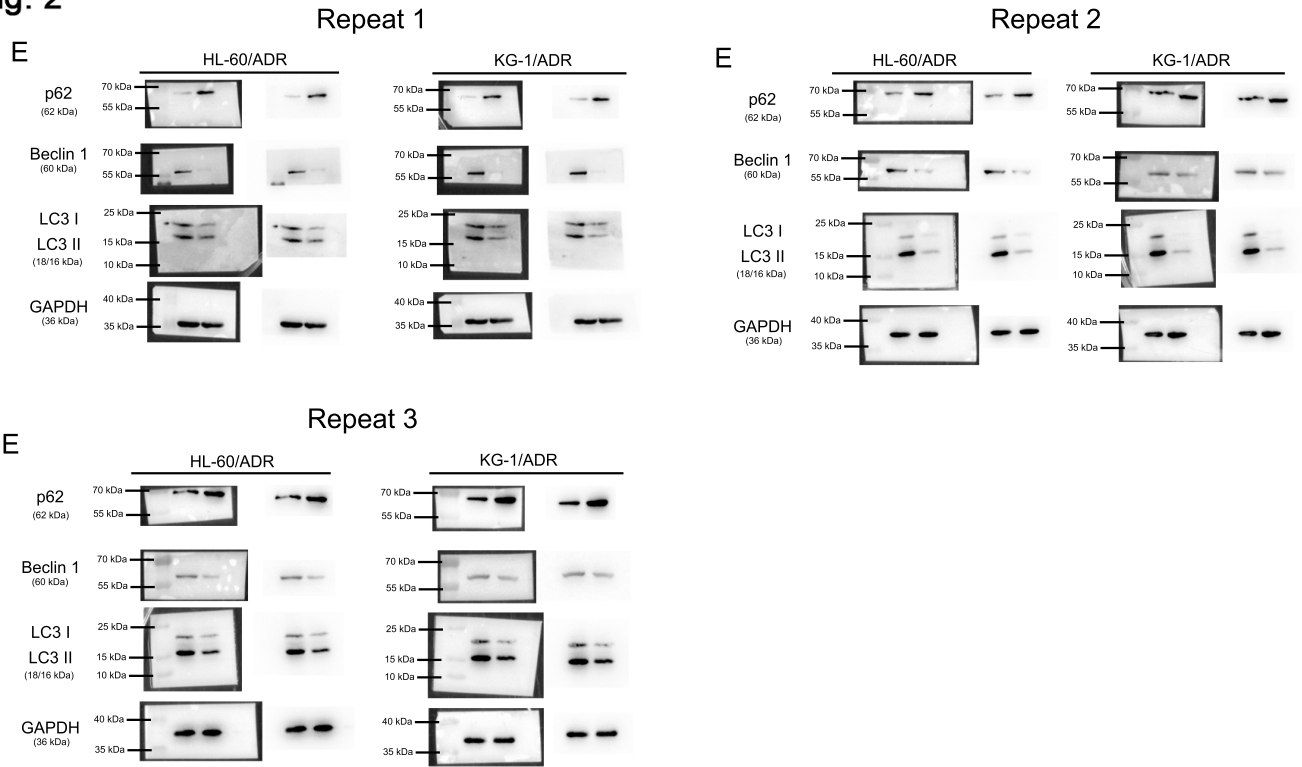

Fig. 3

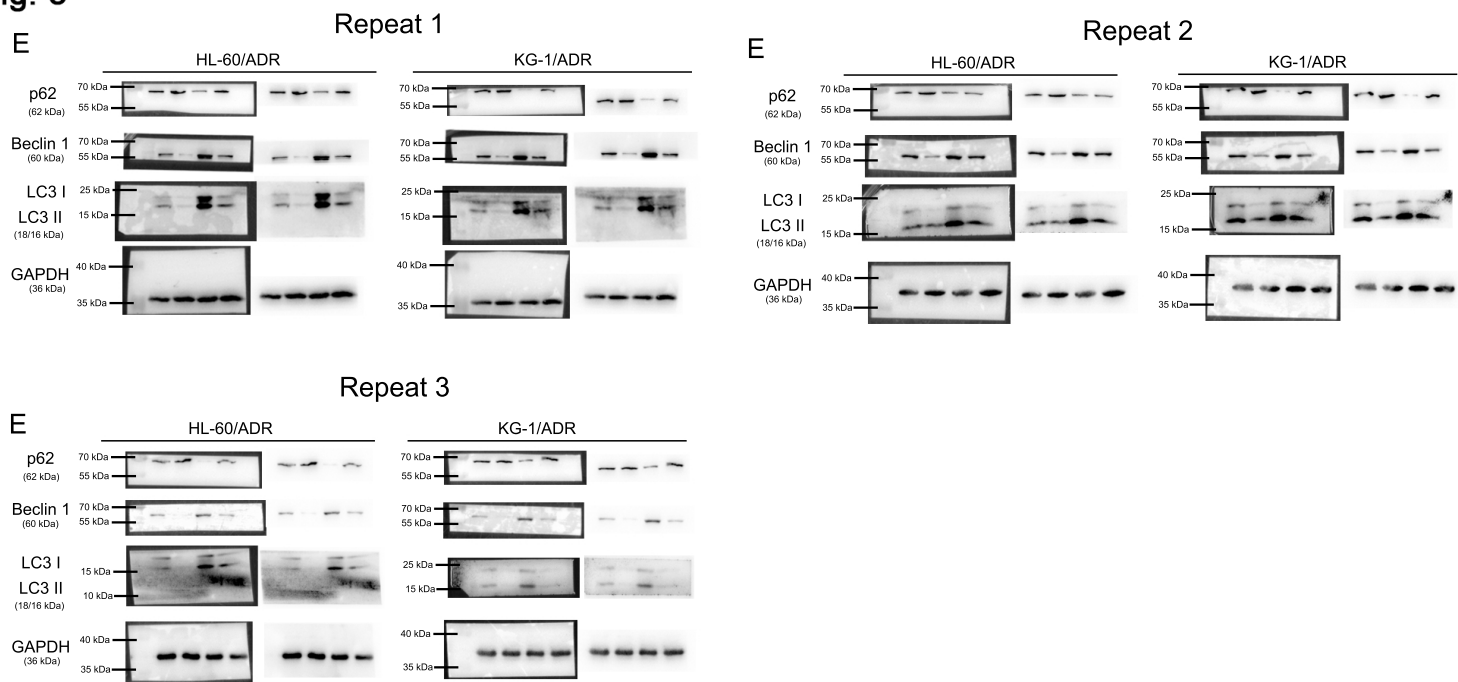

Fig. 4

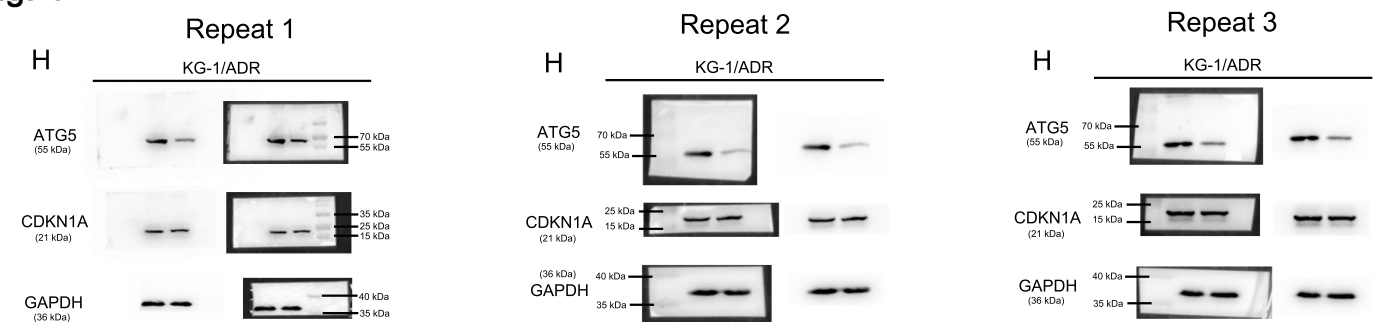

Fig. 5

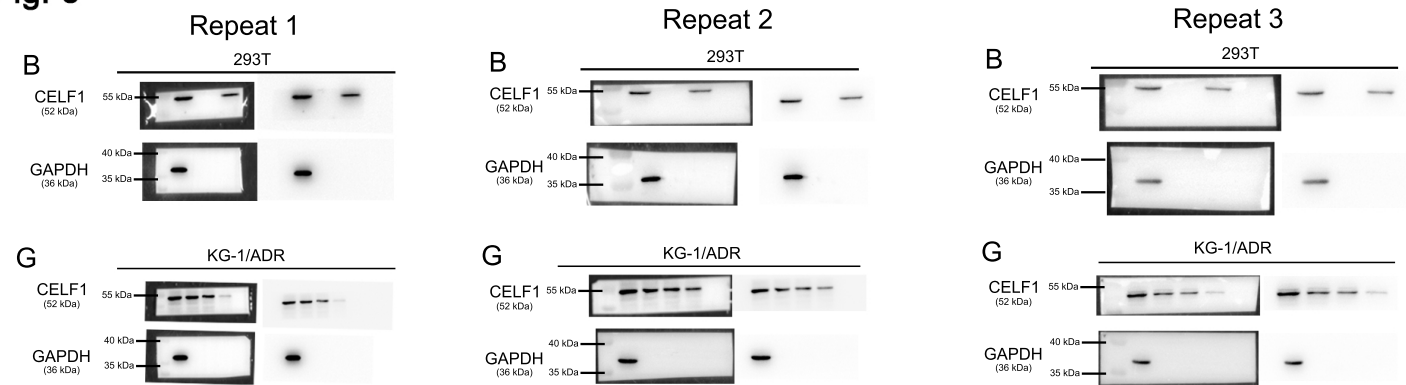

Fig. 6

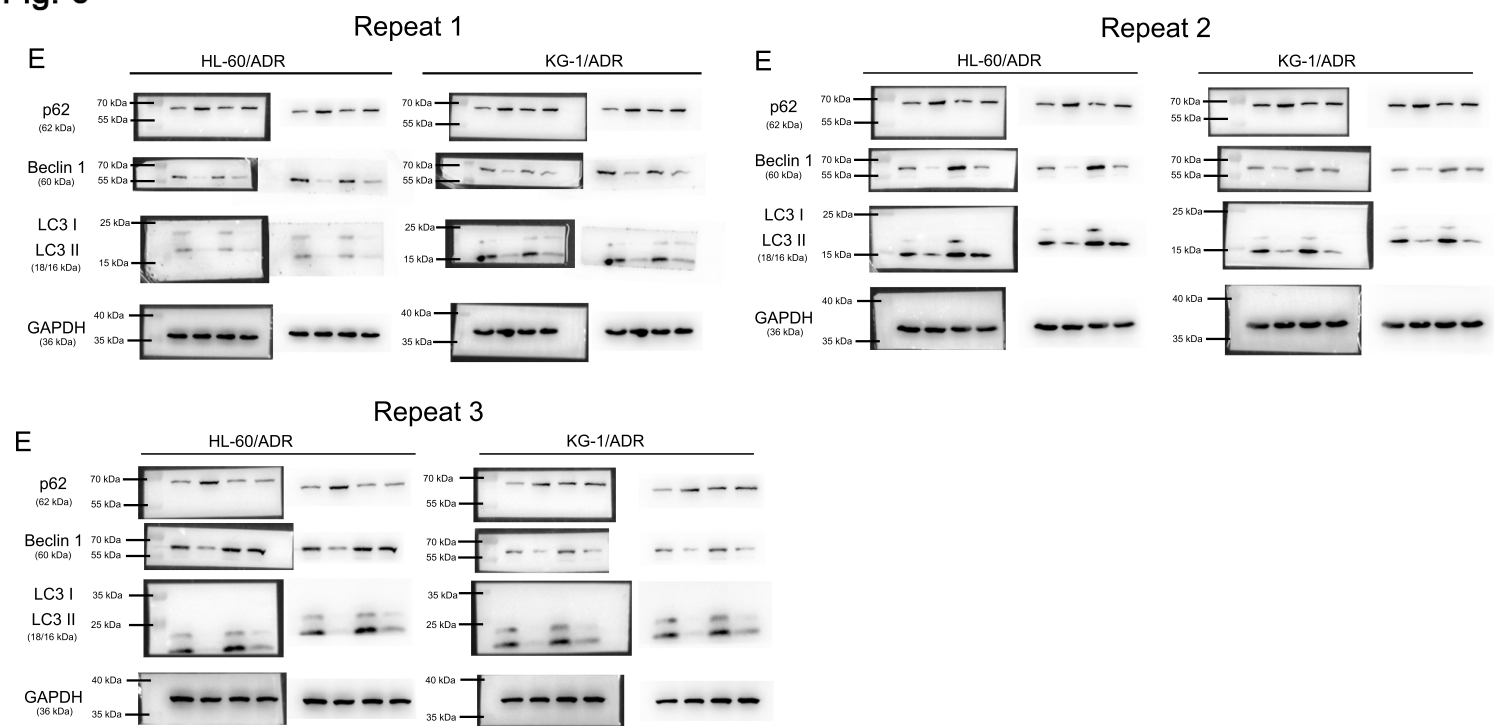

Fig. S1

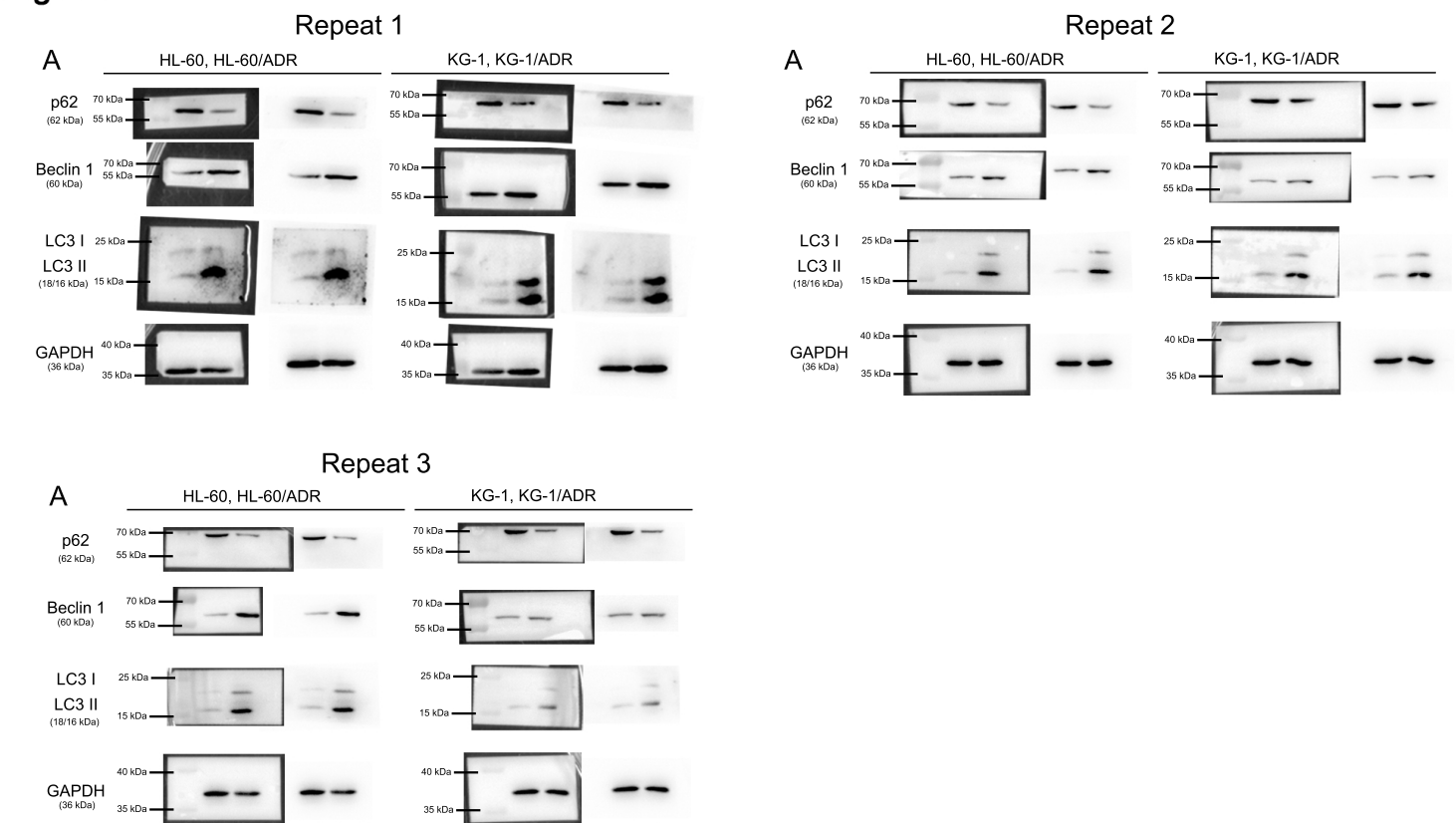

**Fig. S1**

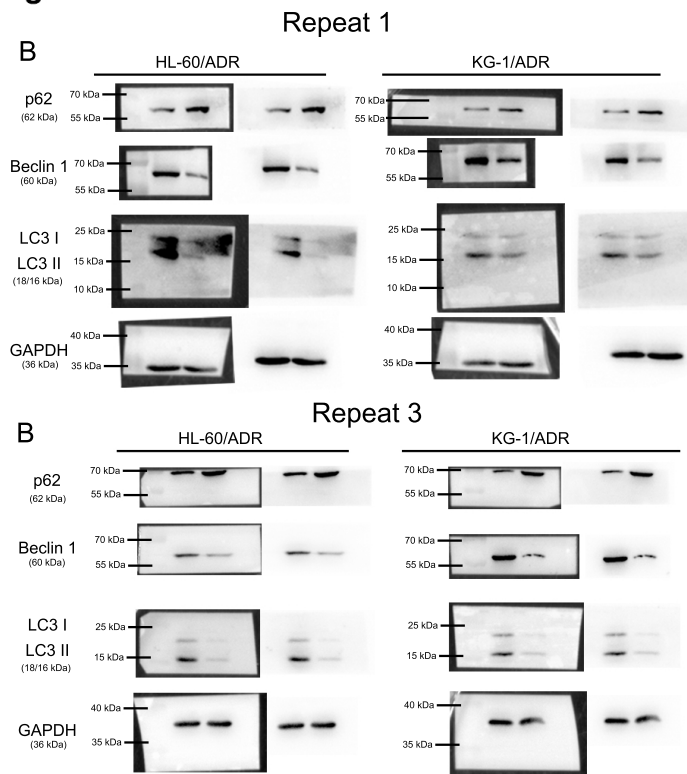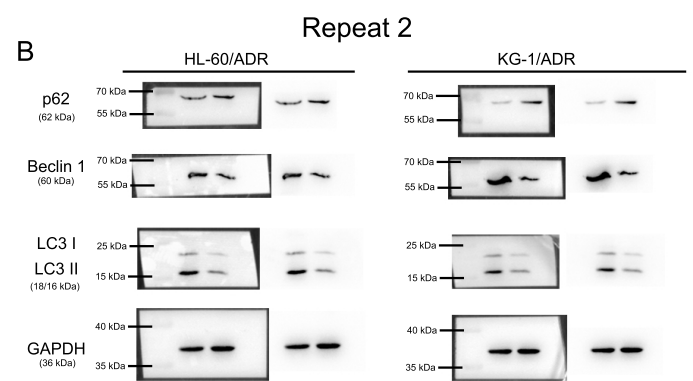

**Fig. S2**

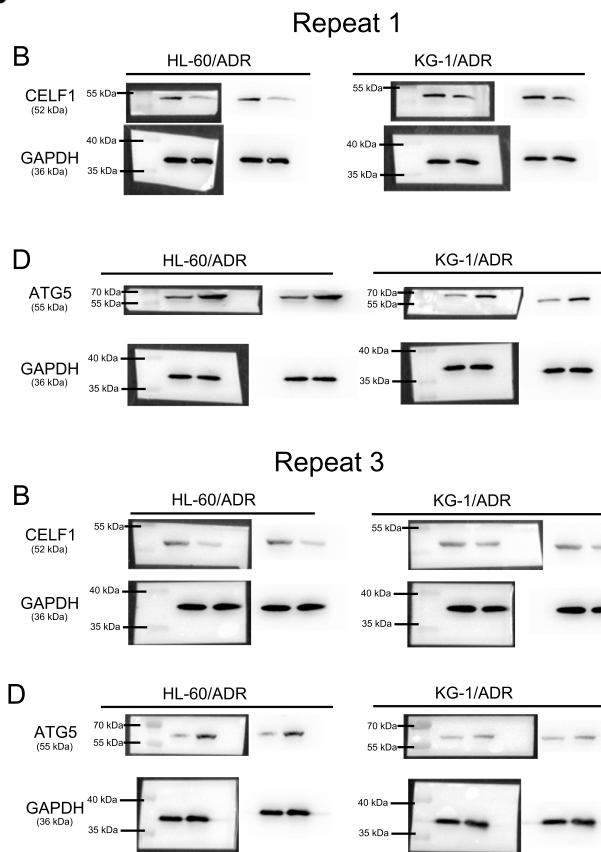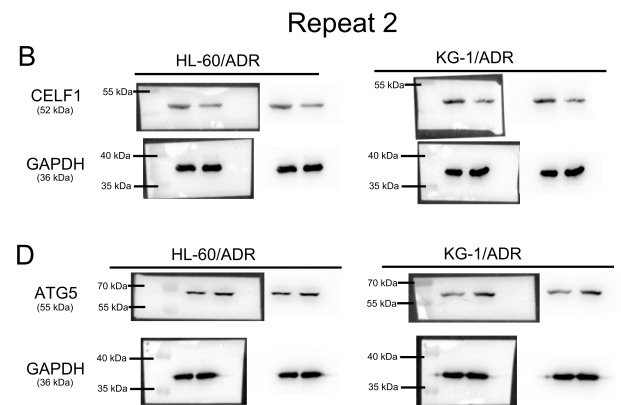

Fig. S3

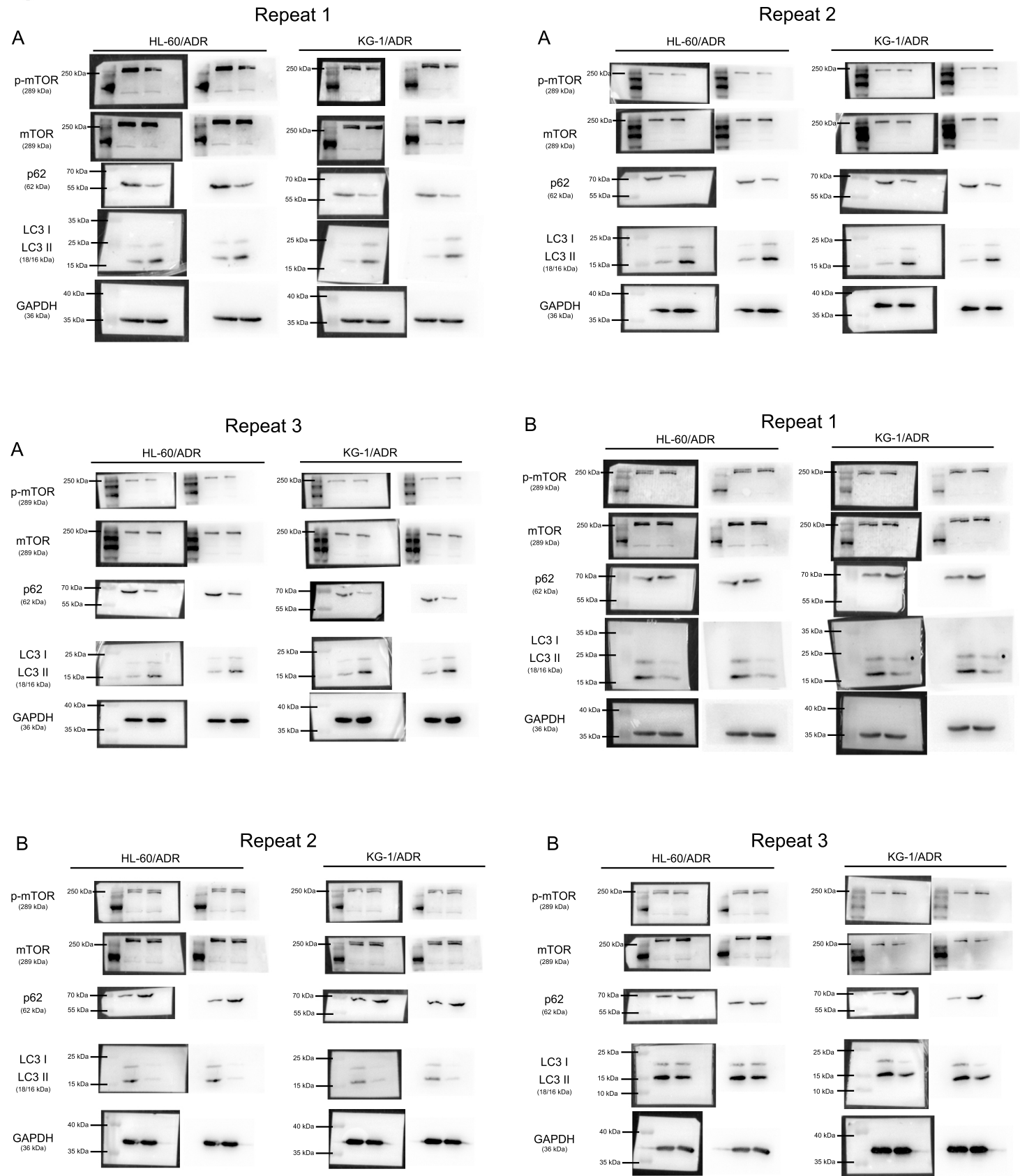

Supplement: Supplementary file 3 — Original data files of Western blot [file 41419_2025_7926_MOESM3_ESM.pdf]
